# The *Agrobacterium fabrum* efflux pump PecM is produced in response to the plant exudate 4-hydroxybenzaldehyde to avoid disruption of central metabolism

**DOI:** 10.1128/jb.00150-25

**Published:** 2025-06-13

**Authors:** Arpita Ghosh, Anne Grove

**Affiliations:** 1 Department of Biological Sciences, Louisiana State University 124526, Baton Rouge, Louisiana, USA; Philipps-Universitat Marburg Fachbereich Biologie, Marburg, Germany

**Keywords:** gene regulation, 4-hydroxybenzoate, metabolomics, PecS, transcription factor

## Abstract

*Agrobacterium fabrum* is a phytopathogen that causes crown gall disease. In the rhizosphere, it encounters plant exudates, some of which are toxic, such as 4-hydroxybenzaldehyde (4HBA). Others, including 4-hydroxybenzoate (4HB), participate in the induction of virulence genes. *A. fabrum* encodes the transcription factor PecS, which has been reported to enhance bacterial fitness in the rhizosphere. The gene encoding PecS is divergent from *pecM*, which encodes an efflux pump. PecS represses both *pecS* and *pecM*, as evidenced by increased expression in the presence of the PecS ligand urate and by elevated *pecM* expression in a *pecS* disruption strain. We report here that the expression of *pecM* is induced selectively by 4HBA. Expression of genes encoding enzymes involved in the degradation of 4HB is induced by both 4HBA and 4HB, as expected; however, overexpression of *pecM* attenuates the induction by 4HBA, suggesting that 4HBA is a substrate for PecM. Consistent with this inference, untargeted metabolomics shows that 4HBA accumulates intracellularly when *pecM* is disrupted. Analysis of PecS by thermal stability assay and DNase I footprinting suggests that 4HBA is not a ligand for PecS. Taken together, our data suggest that 4HBA is a substrate for PecM.

**IMPORTANCE:**

Plant roots secrete a number of compounds that may be toxic to bacteria residing in the surrounding soil. One such bacterium is *Agrobacterium fabrum*, which infects plants and induces tumor formation. We show here that an *A. fabrum* strain in which the efflux pump PecM has been disrupted accumulates 4-hydroxybenzaldehyde, and that this plant root exudate induces the expression of *pecM*. Our data suggest that PecM and PecS, a transcription factor that regulates *pecM* expression, both function to promote *A. fabrum* fitness in the rhizosphere. As a competitive advantage in the rhizosphere is a prerequisite for subsequent plant infection, our data contribute to a more complete understanding of the *A. fabrum* infection process.

## INTRODUCTION

Phytopathogens are responsible for great losses in the agricultural industry because they can subvert or evade the host-mediated defenses and induce expression of genes associated with bacterial fitness and virulence (1). In the rhizosphere, the phytopathogens encounter a variety of organic compounds secreted by plants or other bacteria. The plant root exudate is a mixture of a wide assortment of organic compounds, including primary and secondary metabolites, where the primary metabolites, such as carbohydrates, amino acids, and organic acids, are secreted in larger quantities compared to the secondary metabolites, such as auxins and flavonoids (2). In addition, abiotic stress conditions can alter the composition of root exudates and possibly root microbiota, as exemplified by the recent report that xanthine secreted by salt-stressed soybean attracts beneficial *Pseudomonas* species (3). Compounds found in root exudate also include phenolic compounds and aromatic hydrocarbons such as coumarate, vanillate, 4-hydroxybenzaldehyde (4HBA), and 4-hydroxybenzoate (4HB) (4, 5). Soil saprophytes chemotax toward these plant exudates, which signal proximity to a susceptible plant host (6). In *Pseudomonas putida*, for example, uptake of 4HB through the transporter PcaK promotes chemotaxis by leading to production of the chemoreceptor PcaY (7). *Agrobacterium fabrum* has also been reported to chemotax toward 4HB, which subsequently contributes to the induction of virulence genes (8).

**Fig 1:**
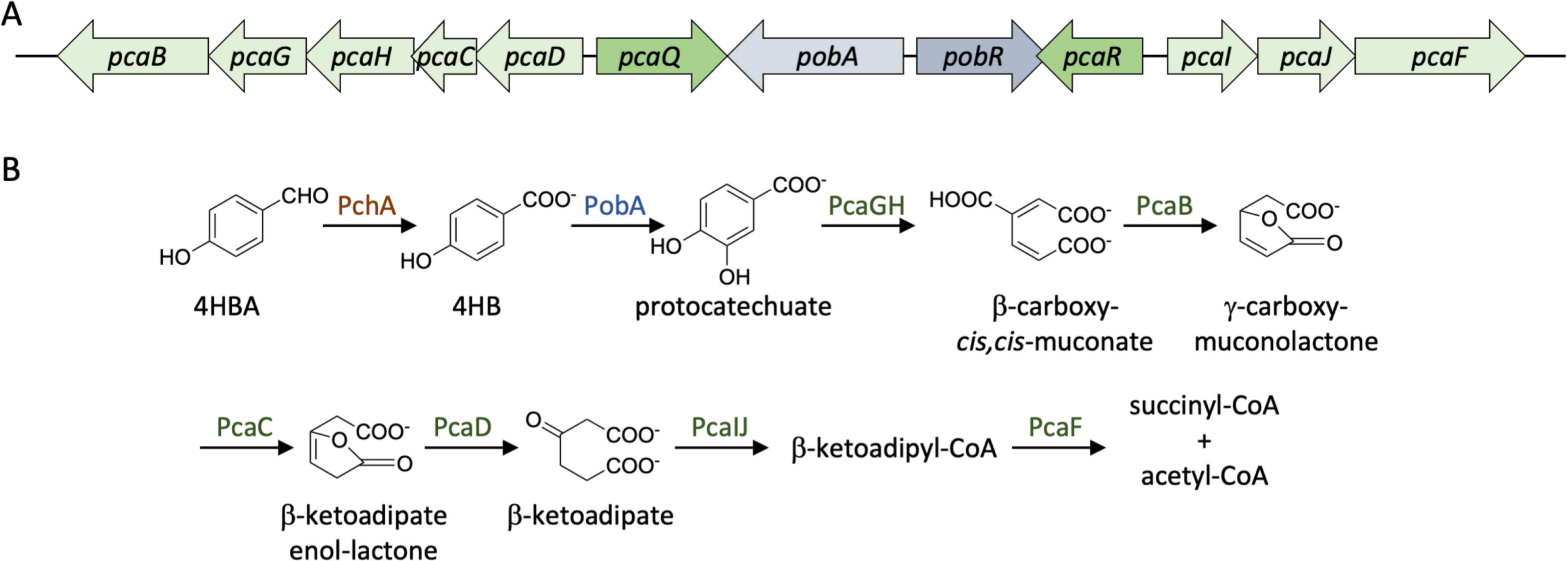
Degradation of 4-hydroxybenzaldehyde (4HBA). (A) *A. fabrum* genomic locus encoding enzymes and transcription factors associated with 4HB degradation. Genes encoding transcription factors are identified by darker shading. (B) Pathway for degradation of 4HBA. The enzyme that converts 4HBA to 4HB (PchA) has not been identified in *A. fabrum*.

Aromatic compounds found in the soil are degraded mostly by aerobic and anaerobic bacteria and aerobic fungi (9). These aromatic compounds, either in the form of aromatic hydrocarbons or phenolic compounds, are converted to catechol (in some bacteria) or protocatechuate (in bacteria and fungi), respectively (10). Separate pathways convert protocatechuate and catechol to β-ketoadipate, which is, in turn, broken down to acetyl-CoA and succinyl-CoA for entry into the citric acid cycle (10). Alternatively, catechol may be cleaved by catechol 2,3-dioxygenases in the meta-cleavage pathway, which ultimately leads to the production of pyruvate and acetaldehyde (11). The β-ketoadipate pathway for aromatic compound degradation is widely distributed in soil bacteria and fungi, including in members of the genus *Agrobacterium* (Fig. 1).

Degradation of 4HB has been characterized in some detail. In a number of bacterial species, such as *Acinetobacter baylyi* and *P. putida*, 4HBA is oxidized to the corresponding benzoate (i.e., 4HB) by 4-hydroxybenzaldehyde dehydrogenase, annotated as PchA (10, 12). In *Agrobacterium* spp., the corresponding enzyme has not been identified. PobR is an activator of *pobA*, which encodes 4-hydroxybenzoate hydroxylase, the enzyme that catalyzes the conversion of 4HB to protocatechuate (Fig. 1). In several species, *pobA* expression has been reported to be induced by 4HB, including in *Agrobacterium* spp. The *A. fabrum* gene cluster associated with 4HB degradation encodes two additional transcription factors, PcaR and PcaQ; PcaR activates *pcaIJF*, whereas PcaQ acts as an activator of *pcaDCHGB* genes. β-ketoadipate is the inducer of *pcaIJF* genes (13), while PcaQ responds to two labile intermediates of the protocatechuate pathway, namely β-carboxy-*cis,cis*-muconate and γ-carboxymuconolactone (14).

*A. fabrum* is a Gram-negative soil bacterium that causes crown gall disease in susceptible plants. This organism has also been considered as nature’s genetic engineer due to its ability to execute horizontal gene transfer to a recipient plant that results in tumor formation (15). Transformation mediated by *A. fabrum* is a complex procedure involving mobilization of its transfer DNA (T-DNA) from the Ti plasmid into the plant with the help of a Type IV secretion system, followed by integration into the plant chromosome. Expression of genes encoded on the integrated T-DNA causes uncontrolled proliferation and tumor formation as well as production of sugar derivatives, the so-called opines, which support bacterial growth (16).

**Fig 2:**
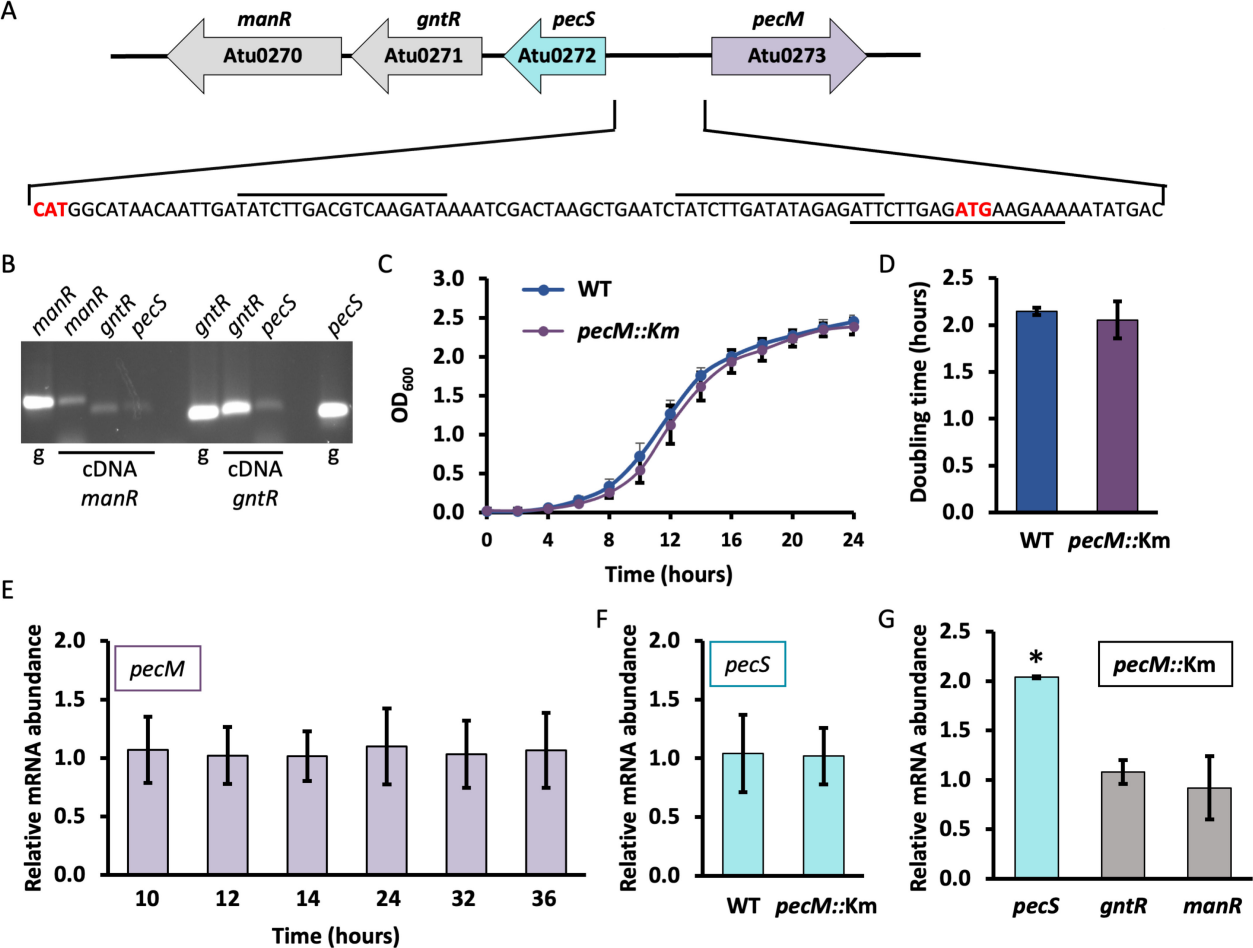
Locus encoding *A. fabrum* PecM and phenotypes associated with inactivation of *pecM*. (A) Genomic locus encoding the divergent genes *pecS* and *pecM*. Genes downstream of *pecS* encode a GntR transcription factor and a mandelate racemase family enzyme. (B) Analysis of a possible operon by RT-PCR. Total RNA was isolated from WT cells grown to mid-log phase in LB media. Templates for PCR reactions are identified below each lane; g denotes genomic DNA, cDNA *manR* is cDNA created with a primer complementary to the *manR* coding region, and cDNA *gntR* is cDNA created with a primer complementary to the *gntR* coding region. The annotation above the lanes identifies amplicons from the respective coding regions. (C and D) Growth curves and doubling times for WT (blue) and *pecM*::Km cells (purple). (E) Relative *pecM* mRNA abundance at the indicated times after inoculation. (F) Relative *pecS* mRNA abundance in WT and *pecM*::Km cells during exponential growth. (G) Relative *pecS, gntR*, and *manR* mRNA abundance in *pecM*::Km cells during stationary phase. Asterisk reflects statistically significant difference compared to the mRNA abundance in WT cells based on a Student’s t-test; *P < 0.05. Data in panels C–G reflect the mean ± SD from three biological replicates.

*A. fabrum* encodes the transcription factor PecS, which belongs to the MarR (multiple antibiotic resistance regulator) family (17, 18). *A. fabrum* PecS has been inferred to confer fitness during the transition from the rhizosphere to the plant host, as evidenced by phenotypes associated with inactivation of *pecS*; however, it is not required for virulence (19). The gene encoding PecS is divergently oriented from *pecM*, which encodes an efflux pump (Fig. 2A), and PecS represses the expression of both *pecS* and *pecM* by binding to both *pecS* and *pecM* promoters (18, 19). *A. fabrum* PecS responds to the purines urate and xanthine, with xanthine selectively inducing *pecS,* while urate induces expression of both *pecS* and *pecM*. The substrate for *A. fabrum* PecM is unknown. The divergent genes encoding PecS and PecM appear to have been distributed by horizontal gene transfer, leading to integration of PecS into existing gene regulatory networks, with PecS function apparently evolving to suit the regulatory needs of the individual bacterium (20). PecS was originally described in the necrotrophic plant pathogen *Dickeya dadantii*, where it was shown to be a critical regulator of virulence genes, including genes encoding enzymes involved in the maceration of the plant cells. The inducing ligand for *D. dadantii* PecS has not yet been reported (21). *D. dadantii* PecM exports the blue-pigmented antioxidant indigoidine, which contributes to virulence (22). Since *A. fabrum* does not encode the indigoidine biosynthetic genes, the substrate for *A. fabrum* PecM is bound to be different. Here, we report that 4HBA induces the expression of *A. fabrum pecM*. The patterns of gene expression and untargeted metabolomics data suggest that 4HBA is a substrate for PecM, and that PecM is required to avoid disruption of central metabolism during degradation of 4HBA.

## RESULTS

### Disruption of *pecM* is associated with increased *pecS* expression

As per MicrobesOnline (https://microbesonline.org), the *pecS* gene is predicted to be part of an operon also encoding Atu0271 (a GntR family transcription factor) and Atu0270 (a mandelate racemase family protein) (Fig. 2A). To test whether *Atu0272-Atu0270* constitutes an operon, we performed reverse transcriptase PCR using RNA isolated from an exponential phase culture. cDNA generated using a reverse primer complementary to *Atu0270* (*manR*) was used to perform PCR with primers that will specifically amplify *Atu0271* (*gntR*) or *Atu0272* (*pecS*) fragments only if those three genes are part of the same operon. Primers amplifying *manR* and *gntR* yielded PCR products of comparable intensity, whereas the product obtained with *pecS*-specific primers was less abundant (Fig. 2B, left lanes). When cDNA was generated with a primer complementary to *gntR*, the product obtained with *pecS*-specific primers was again less abundant (Fig. 2B, right lanes). Since all primer pairs amplified products of comparable abundance using genomic DNA as template (lanes marked g), these data suggest that a polycistronic transcript containing *pecS-gntR-manR* mRNA is indeed produced, but that a separate mRNA may originate from a promoter in the 111 bp *gntR-pecS* intergenic region.

The gene encoding *pecM* was disrupted by Campbell-type insertion of plasmid DNA to generate the mutant strain *pecM*::Km. To investigate the effect of disruption of *pecM*, WT and *pecM::*Km cells were grown to stationary phase at 28°C in LB medium. The generation time for both strains was similar, which implies that disruption of the *pecM* gene has no significant effect on the growth of *A. fabrum* in rich media (Fig. 2C and D). The relative abundance of the *pecM* transcript level in the WT strain showed that the expression of *pecM* is not growth phase specific during growth in rich media, as the transcript level remained the same during exponential growth and in the stationary phase (Fig. 2E). Since *pecM* is repressed by the purine-responsive PecS, this observation also implies that ligands for PecS do not accumulate differentially under these conditions.

In *D. dadantii*, it was shown that PecM is necessary for achieving full repressor activity of PecS, as cells in which *pecM* is disrupted express more PecS (23). To determine whether a similar scenario applies for *A. fabrum*, we tested the relative abundance of *pecS* transcript level in the exponential phase in WT and *pecM*::Km mutant strains. The results show that *pecS* mRNA abundance did not change in the *pecM::*Km mutant strain compared to WT during exponential growth (Fig. 2F), indicating that PecM is not required for PecS to function as a repressor. However, a modest yet significant increase in *pecS* transcript level was observed during the stationary phase in the *pecM*::Km mutant strain compared to WT (Fig. 2G). The simplest interpretation of this observation is that the accumulation of a PecS ligand occurs in the stationary phase in the absence of PecM, possibly because the PecS ligand is a substrate for the efflux pump PecM. No accumulation of the *gntR* or *manR* transcripts was observed (Fig. 2G), consistent with the inference that most of the *gntR* transcript initiates downstream of *pecS*.

### Increased *pecS* expression in *pecM*::Km cells is not due to the accumulation of purines

**Fig 3:**
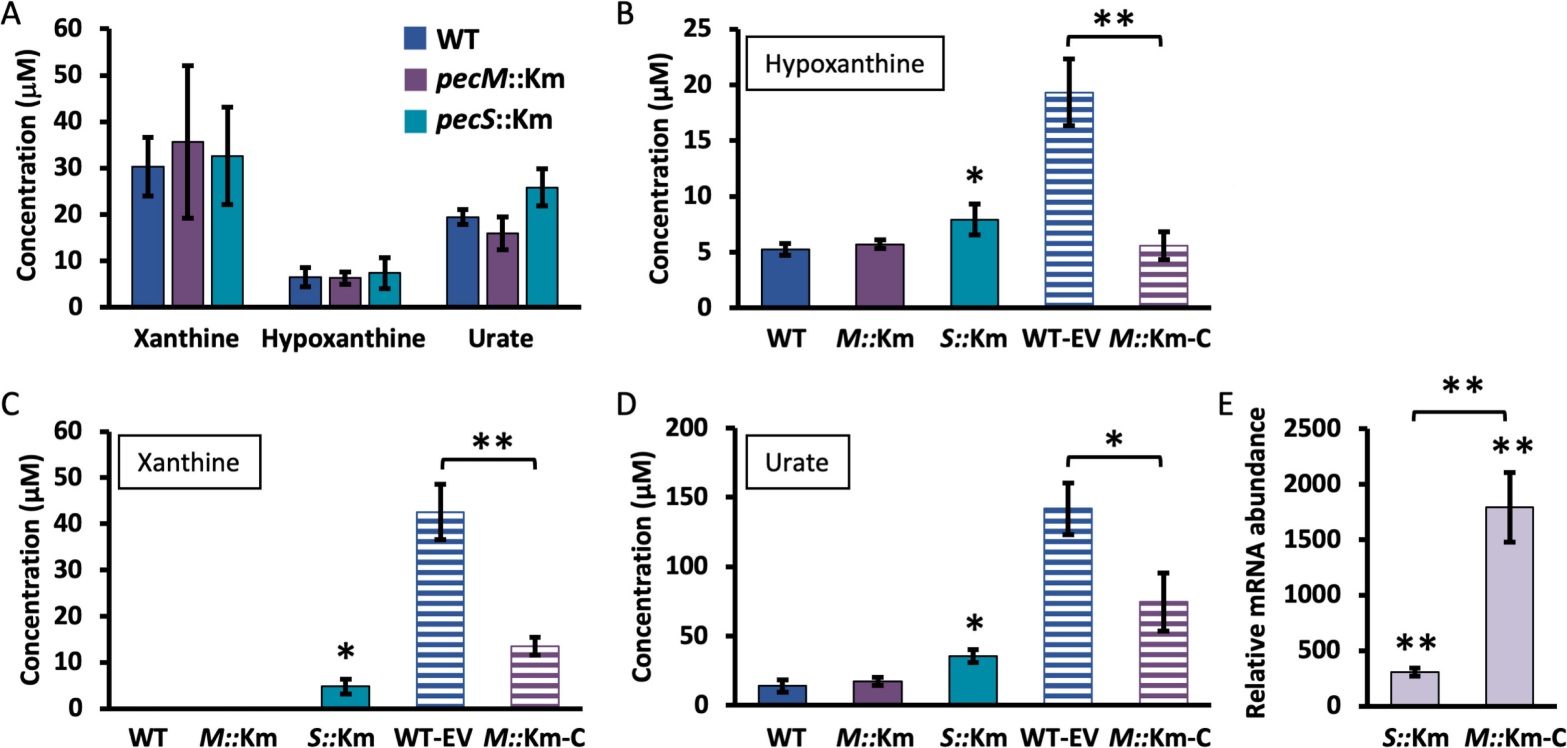
Cellular abundance of purines. (A) The identified purines were quantified using the Amplex Red assay during exponential growth in WT (blue), *pecM*::Km (purple), and *pecS*::Km (cyan) cells. (B and D) Hypoxanthine (**B**), xanthine, (**C**) and urate (**D**) levels were measured in the stationary phase in the identified strains, WT, *pecM*::Km (*M*::Km-C), *pecS*::Km (*S*::Km), WT-EV, and *pecM*::Km-C (*M*::Km-C). (E) Expression of *pecM* in *pecS*::Km (*S*::Km) and *pecM*::Km-C (*M*::Km-C) during exponential growth. Data reflect the mean ± SD from three biological replicates. Asterisks reflect statistically significant differences based on a Student’s t-test; *P < 0.05; **P < 0.001).

*A. fabrum* PecS binds the ligands urate and xanthine with a Kd ~9 μM, with urate inducing expression of both *pecS* and *pecM*, while xanthine selectively induces expression of *pecS* (18, 19). We therefore used Amplex Red assays to quantify the levels of urate, xanthine, and hypoxanthine during exponential growth and in the stationary phase to determine whether purine accumulation would explain the increased *pecS* expression observed in the stationary phase in *pecM*::Km cells compared to WT. In this assay, we also included cells in which *pecS* was disrupted (*pecS*::Km) (19). Hypoxanthine is not a ligand for PecS and was included as a control. In this linked enzymatic assay, oxidation of the purine is associated with the production of H_2_O_2_, which, in turn, reacts stoichiometrically with the Amplex Red reagent to produce the fluorescent resorufin. As shown in Fig. 3A, there was no difference in the accumulation of urate, xanthine, or hypoxanthine between WT, *pecS*::Km, and *pecM*::Km cells in exponential phase, which is consistent with the unaltered relative expression of *pecS* in *pecM*::Km (Fig. 2F). In stationary phase, a slight increase in the accumulation of purines was observed for *pecS*::Km but not in *pecM*::Km cells, which suggests that the increased relative abundance of *pecS* mRNA in *pecM*::Km stationary phase cells (Fig. 2G) was not due to the accumulation of either of the known PecS ligands, urate or xanthine (Fig. 3B through D, solid bars). This suggests the existence of another inducer of *pecS* accumulating in *pecM*::Km stationary phase cells, possibly because it is a substrate for PecM.

We also performed this assay using the strain in which the *pecM* disruption is complemented with *pecM* expressed from a plasmid (strain denoted *pecM*::Km-C), as well as controls in which WT and *pecM*::Km cells harbor the empty vector (denoted EV). These cultures were grown with chloramphenicol (Cm; 60 μg mL^−1^) as the plasmids encode Cm resistance. Surprisingly, a significant increase in the accumulation of most purines was evident for these strains (Fig. 3B through D, striped bars). Accumulation of purines was greater in WT-EV compared to *pecM*::Km-C cells, possibly because overproduction of PecM in the *pecM*::Km-C strain resulted in expulsion of some of the antibiotic. That *pecM* is overexpressed in *pecM*::Km-C cells was verified by measuring *pecM* mRNA levels in *pecM*::Km-C, which revealed almost 10-fold greater mRNA accumulation compared to the already elevated level detected in *pecS*::Km (Fig. 3E; samples collected during exponential growth).

**Fig 4:**
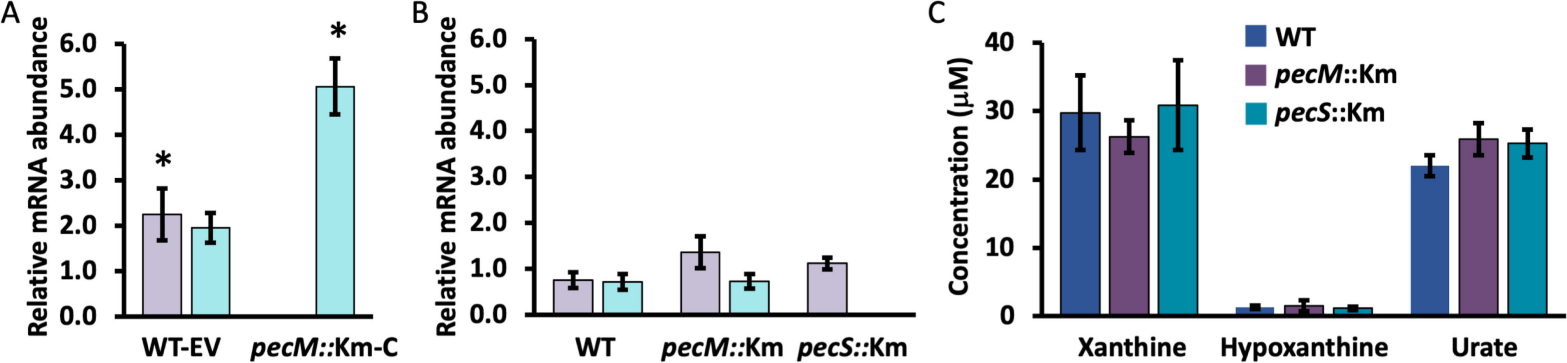
Effects of chloramphenicol (Cm). (A) Expression of *pecM* (lavender) and *pecS* (cyan) in the identified strains grown to exponential phase with 60 μg mL^−1^ Cm. *pecM* gene expression was not measured in *pecM*::Km-C cells. (B) Expression of *pecM* (lavender) and *pecS* (cyan) in the identified strains during exponential growth in response to 15 μg mL^−1^ Cm. (C) Intracellular purine levels as determined by the Amplex Red assay during exponential growth in WT (blue), *pecM*::Km (purple), and *pecS*::Km (cyan) cells grown with 15 μg mL^−1^ Cm. Data reflect the mean ± SD from three biological replicates. Asterisks reflect statistically significant differences compared to unsupplemented cultures based on a Student’s t-test; *P < 0.05).

The observation that purines accumulate in strains WT-EV and *pecM*::Km-C as measured in stationary phase (Fig. 3B through D) predicts that *pecS* and *pecM* expression should be elevated in these strains. Indeed, expression of *pecS* and *pecM* was modestly increased during exponential growth in WT-EV grown with 60 μg mL^−1^ of Cm (Fig. 4A). In addition, a further increase in the expression of *pecS* was observed in *pecM::*Km*-*C compared to EV. Since the accumulation of purines was lower in the complemented strain compared to WT-EV (Fig. 3B through D), this observation was unexpected. This finding raises the possibility of the accumulation of another inducer for the *pecS* gene. By comparison, growth with 15 μg mL^−1^ Cm (which does not impose a growth phenotype, most likely on account of its efficient inactivation by chloramphenicol acetyl transferase) did not result in elevated expression of *pecS* or *pecM* (Fig. 4B) or in altered purine levels (Fig. 4C).

### PecM may contribute modestly to chloramphenicol export

Considering that *pecM*::Km-C cells, which overexpress *pecM*, accumulate less of the purines compared to WT-EV (Fig. 3B through D), we also investigated the effects of Cm in WT cells and cells in which either *pecS* or *pecM* were disrupted. The biological relevance of responses to Cm is that resistance to Cm would be protective in a soil environment where other bacterial species, such as *Streptomyces venezuelae* and other *Streptomyces* species, secrete Cm. Cells were grown to OD_600_ ~0.75, followed by treatment with sublethal Cm (15 µg mL^−1^) for 45 min. We examined the relative expression of genes predicted to be associated with Cm sensitivity, with the following expectation: if PecM has a physiological role in Cm export, then cellular Cm levels may be lower when *pecM* is overexpressed and higher when *pecM* is disrupted. Accordingly, the expression of Cm-sensitive genes should be higher in *pecM*::Km cells compared to WT. As detailed in Fig. S1, expression of several genes did follow this general trend, suggesting that the absence of *pecM* is associated with increased accumulation of Cm and hence modestly elevated expression of Cm-sensitive genes.

Taken together, these gene expression data lead us to one inference and one convenient tool for further exploration of substrates for PecM: (i) Since expression of Cm-responsive genes is consistently, albeit modestly, increased when PecM is absent, it is possible that PecM can participate in the export of Cm. We suspect that this contribution is of marginal significance *in vivo*, where induction of chloramphenicol acetyl transferase (*catB*) is undoubtedly the primary means of ensuring Cm resistance. (ii) Since *pecM* is induced ~2-fold in the WT-EV strain grown with Cm (Fig. 4A), the expectation would be for this strain to accumulate less of the physiologically relevant PecM substrate compared to WT.

### 4-hydroxybenzaldehyde (4HBA) accumulates in *pecM*::Km cells

**Fig 5:**
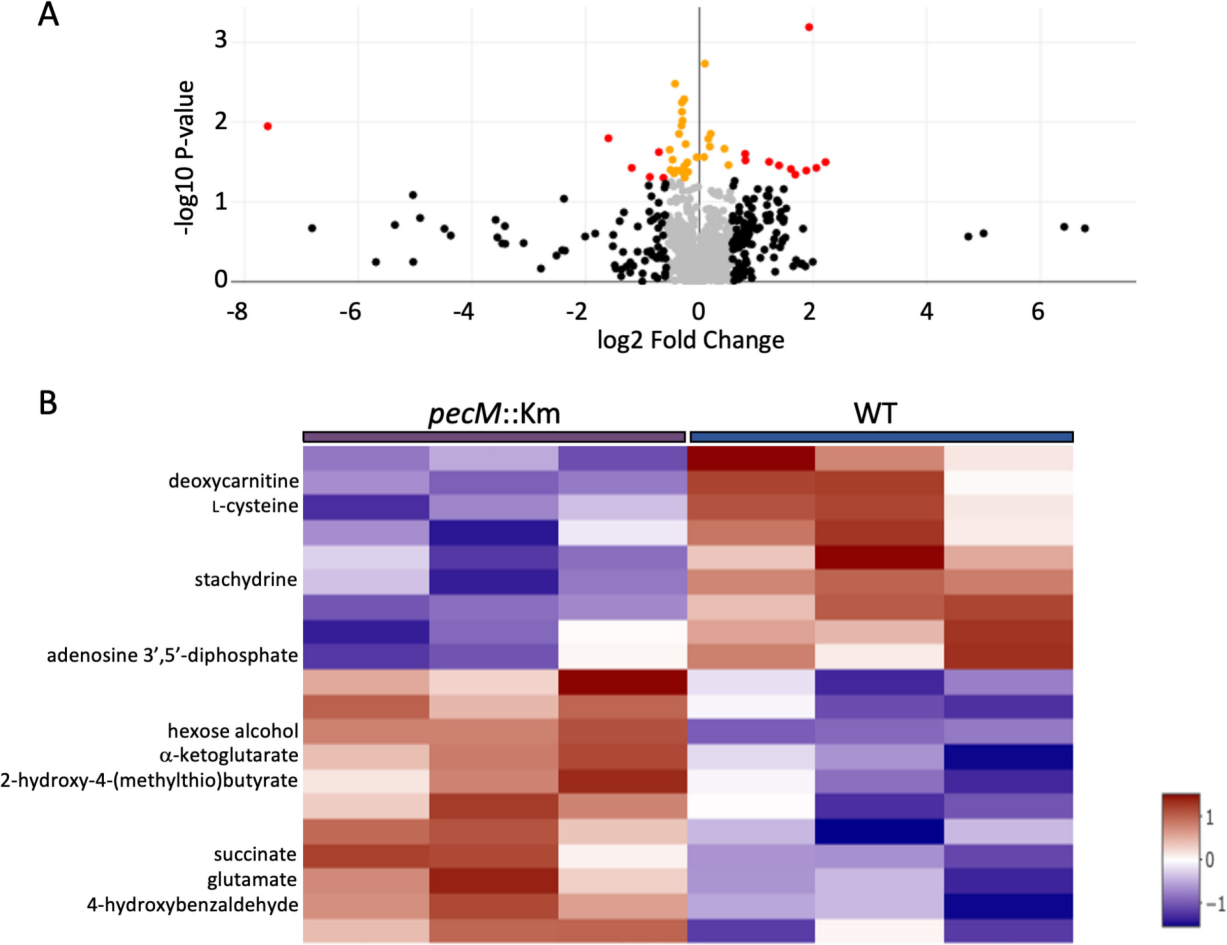
Differentially accumulating metabolites in *pecM*::Km cells compared to WT in the stationary phase. (A) Volcano plot reflecting differentially accumulating metabolites. Features marked with red dots meet both the fold-change of |1.5| and statistical significance, *P* < 0.05. Featured marked with yellow dots meet the statistical significance, but not the fold-change cutoff. Features marked with black dots meet the fold-change cutoff, but not statistical significance. (B) Heatmap of differential accumulation of metabolites. Red indicates higher expression, and blue indicates lower expression. Features identified with confidence are identified at the left.

A significant increase in the expression of *pecS* was observed for *pecM*::Km cells grown to stationary phase (Fig. 2G), implying accumulation of an inducer for the *pecS* gene. Such accumulation would be expected if the inducer were a substrate for PecM. We therefore performed untargeted metabolomics using cells grown to stationary phase (36 h post-inoculation). Three biological replicates each of WT, and *pecM*::Km strains were used. To capture global metabolomics profiles of WT and *pecM*::Km strains, LC-MS conditions specific for positive and negative ionization with a specific mass resolution mode for untargeted screening, confirmation, and quantification of positively and negatively charged metabolites were used. Metabolites were identified based on an internal library, and only features identified with confidence are reported here. Designation of differentially accumulating metabolites was based on an adjusted *P*-value of 0.05 and a fold-change of |1.5|. The Volcano plot illustrates metabolites that meet both thresholds (Fig. 5A; red dots). The heatmap includes the metabolites with the greatest change between strains (Fig. 5B), reflecting qualitatively consistent changes for the triplicate samples; only compounds identified with confidence are labeled.

Among the most highly accumulating, confidently assigned metabolites in *pecM*::Km cells were α-ketoglutarate (4.6-fold), hexose alcohol (3.8-fold), succinate (2.6-fold), and 4HBA (1.7-fold). It is to be noted that this list did not include purine metabolites, which is consistent with our findings from the Amplex assay (Fig. 3). These data suggest these compounds as candidates to be substrates for PecM. We decided to focus on 4HBA, as it is a toxic compound that the bacteria may encounter in the rhizosphere (24). All identified features, whether differentially accumulating or not, are provided in Table S1.

### Gene expression patterns suggest 4HBA is a substrate for PecM

**Fig 6:**
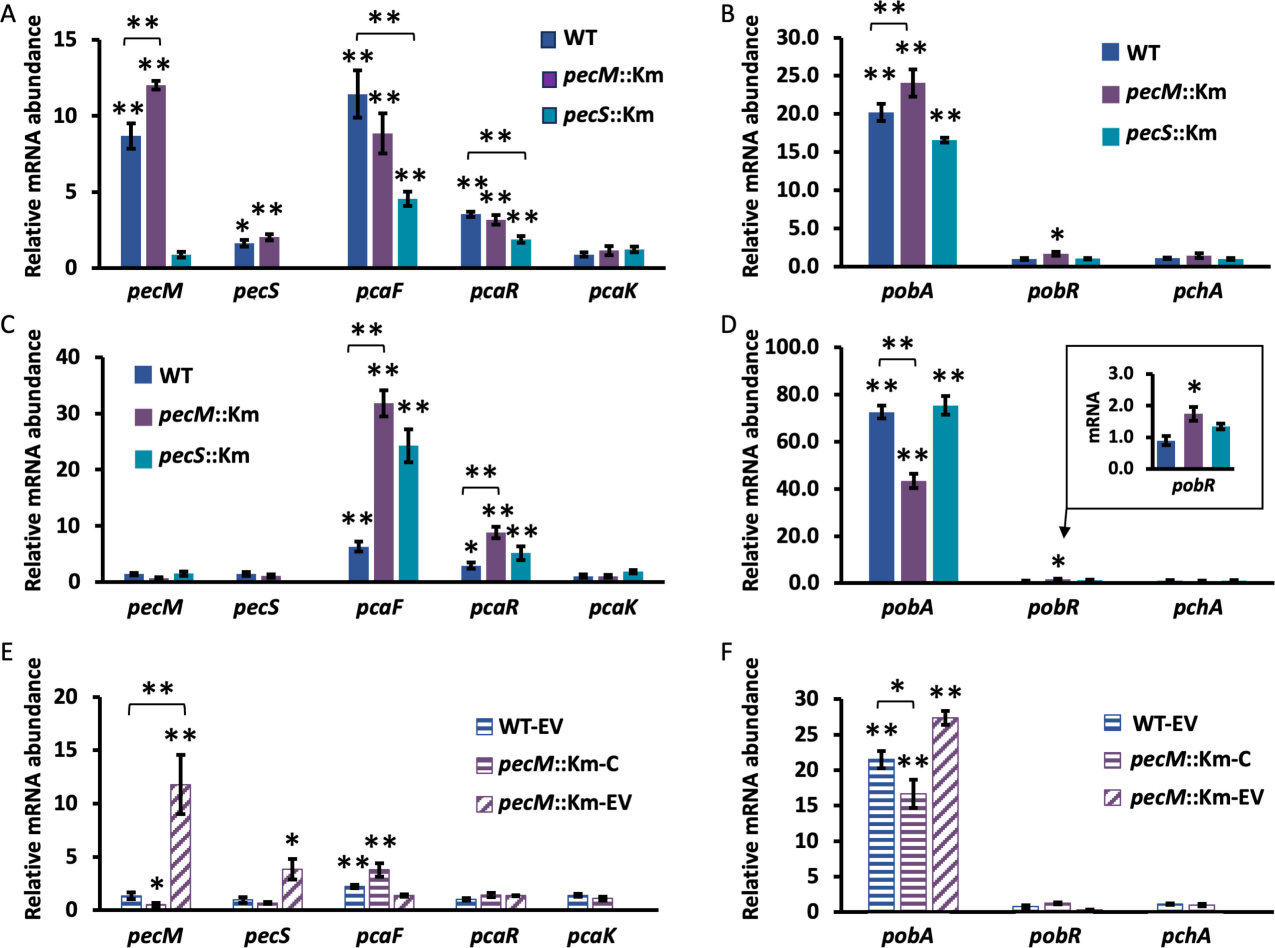
Gene expression in response to 4-hydroxybenzaldehyde (4HBA) or 4-hydroxybenzoate (4HB). (A and B) Gene expression in the identified strains in response to 275 μg mL^−1^ 4HBA during exponential growth. (C and D) Gene expression in the identified strains in response to 275 μg mL^−1^ 4HB during exponential growth. (E and F) Gene expression in the identified strains in response to 333 μg mL^−1^ 4HBA during exponential growth. Data reflect the mean ± SD from at least three biological replicates. Asterisks reflect statistically significant differences compared to unsupplemented cultures unless identified otherwise; based on a Student’s t-test; *P < 0.05; **P < 0.001).

Based on the previous findings that *A. tumefaciens* A348 is chemotactic toward 4HB and because 4HB also participates in the induction of *vir* genes (8), we explored the ability of both 4HBA and 4HB to induce gene expression. In addition, degradation of 4HBA starts with its conversion to 4HB (Fig. 1), thus genes induced by both 4HBA and 4HB would be responding to 4HB or a downstream metabolite. Cells were grown to OD_600_ ~0.75, followed by incubation with either 4HB or 4HBA for 45 min. As shown in Fig. 6A, both *pecM* and *pecS* were significantly induced by 275 μg mL^−1^ 4HBA in WT and *pecM*::Km cells; *pecM* expression represents an amplicon spanning nucleotide positions 32–127, whereas the gene disruption to generate *pecM*::Km occurred between positions 147 and 466 (relative to the 868 nt *pecM* gene). Thus, *pecM* expression in *pecM*::Km reflects the accumulation of an mRNA that does not give rise to functional PecM (possibly affecting its stability). A slightly greater mRNA accumulation was observed in *pecM*::Km cells compared to WT, an outcome consistent with 4HBA being a substrate for PecM. The already elevated *pecM* expression in *pecS*::Km was not further increased on exposure to 4HBA. Two possible (not mutually exclusive) explanations for this observation are that 4HBA is a ligand for PecS (as questioned below) and/or that overexpression of *pecM* in *pecS*::Km leads to efficient export of the inducing ligand 4HBA. No accumulation of the *manR* transcript (Fig. 2A) was observed on exposure to 4HBA (Fig. S2).

PcaF catalyzes the conversion of β-ketoadipyl-CoA to succinyl-CoA and acetyl-CoA (Fig. 1). It is encoded as part of the *pcaIJF* operon, which is under control of PcaR and induced by β-ketoadipate (13). The gene encoding PcaF was comparably induced in WT and *pecM*::Km cells, whereas induction in *pecS*::Km was less pronounced, which could be explained by the overexpression of *pecM* in *pecS*::Km, resulting in export of 4HBA by PecM. While the induction of *pcaR* was more modest compared to that of *pcaF*, it followed the same pattern (Fig. 6A).

The order of structural *pca* genes differs between bacterial species (13). In *P. putida*, the membrane transporter PcaK is encoded as part of the gene cluster *pcaRKF*, and PcaK was shown to participate in the uptake of 4HB (25). The corresponding *pca* gene cluster in *A. fabrum* does not include *pcaK* (Fig. 1). The best BLAST hit to *P. putida* PcaK is Atu2525, which is annotated as a benzoate transport protein (29% identity and 47% homology). *Atu2525* (*pcaK*) was not induced by 4HBA.

The gene encoding PobA, which converts 4HB to protocatechuate (Fig. 1), is controlled by PobR and induced by 4HB (13). As expected, *pobA* was significantly induced by 4HBA, with marginally greater induction in *pecM*::Km compared to WT (Fig. 6B). Induction of *pobR* was only modestly induced in *pecM*::Km cells. PchA, which carries out the conversion of 4HB to 4HBA (Fig. 1), has not been identified in *A. fabrum*. The best BLAST hit to *P. putida,* PchA was Atu4247 (34% identity and 53% homology). *Atu4247* was not induced by 4HBA.

In contrast to the induction seen with 4HBA, treatment with 275 μg mL^−1^ 4HB did not result in the accumulation of *pecM* and *pecS* mRNA in either WT or the two mutant strains (Fig. 6C). This outcome means that 4HBA is the specific inducer; if a downstream intermediate in the degradation pathway were the inducer, comparable induction should have been seen with 4HB. For *pcaF*, a modest increase in mRNA level was observed in WT, with greater induction in the two mutants. For *pcaR*, the level of induction was moderate, but it followed the same pattern as that observed for *pcaF*. Since PcaR responds to β-ketoadipate, the expectation would have been for *pcaF* to be induced similarly or more effectively by 4HB compared to 4HBA, since 4HBA must first be converted to 4HB and ultimately to β-ketoadipate for the induction of *pcaF*. This was not observed in WT cells, only in the two mutant strains, suggesting that part of the 4HB is metabolized by a route that does not include β-ketoadipate. While the greater induction in *pecM*::Km could be explained by accumulation of 4HB because it is a substrate for PecM, leading to more 4HB to be metabolized in the β-ketoadipate pathway, it is unclear why *pcaF* induction would be elevated in *pecS*::Km cells compared to WT. *pcaK* was not induced by 4HB. For *pobA*, 4HB was a much more efficient inducer than 4HBA in all strains (Fig. 6D). This outcome was not surprising; *pobA* is induced by 4HB, and 4HBA would need to be converted to 4HB to induce *pobA* and *pobR*. However, the reduced *pobA* induction in *pecM*::Km cells compared to WT was unanticipated. If 4HB is also a substrate for PecM and accumulates in *pecM*::Km cells, one possibility is that excess 4HB may enter a different degradation pathway, such as its conversion to catechol, resulting in its more rapid depletion. *Atu4247* (putative *pchA*) was not induced by 4HB.

Expression of *pecM* and *pecS* was also examined in *pecM*::Km-C, in which disruption of *pecM* is complemented with *pecM* encoded from a plasmid. Two separate controls for this experiment were included, WT-EV and *pecM*::Km-EV, in which WT and *pecM*::Km strains carry an empty vector. As noted above (Fig. 4A), growth of WT-EV cells leads to increased expression of *pecS* and *pecM*, while very marked overexpression of *pecM* was seen in *pecM*::Km-C (Fig. 3E). In the WT-EV and *pecM*::Km-C strains, induction of *pecM* and *pecS* by 4HBA was no longer seen, even though these cultures were supplemented with a slightly higher concentration of 4HBA (333 μg mL^−1^; Fig. 6E). In WT-EV and *pecM*::Km-C, increased expression of *pecM* (Fig. 4A and 3E) may lead to export of 4HBA, resulting in reduced induction. By contrast, induction of *pecM* and *pecS* in *pecM*::Km-EV (Fig. 6E; cross-hatched bars) was equivalent to that seen in *pecM*::Km (Fig. 6A; purple bars). This indicates that differential induction of gene expression by 4HBA in WT-EV and *pecM*::Km-EV may be ascribed to PecM.

By comparison, induction of *pobA* on addition of 333 μg mL^−1^ 4HBA was similar to that observed in WT exposed to 275 μg mL^−1^ 4HB, also indicating less efficient induction when *pecM* is overexpressed (strain *pecM*::Km-C) and slightly greater induction in *pecM*::Km-EV (Fig. 6F). By contrast, induction of *pcaF* and *pcaR* appears to be compromised by the presence of Cm at the higher concentration (60 μg mL^−1^) used with complementing plasmids (Fig. 6E). PcaR requires β-ketoadipate as an inducer to effect increased gene expression (13). It has been previously reported in *P. putida* that induction of enzymes required for degradation of aromatic compounds is inhibited by Cm, indicating that *de novo* protein synthesis is critical (26). We therefore surmise that the production of β-ketoadipate is compromised when protein synthesis is inhibited by Cm, precluding efficient induction of *pcaF* and *pcaR*.

Taken together, gene expression patterns point to cellular accumulation of 4HBA and enhanced expression of target genes when *pecM* is disrupted, as well as attenuated induction when *pecM* is overexpressed, suggesting that 4HBA is a substrate for PecM. This is consistent with the outcome of the untargeted metabolomics analysis (Fig. 5).

**Fig 7:**
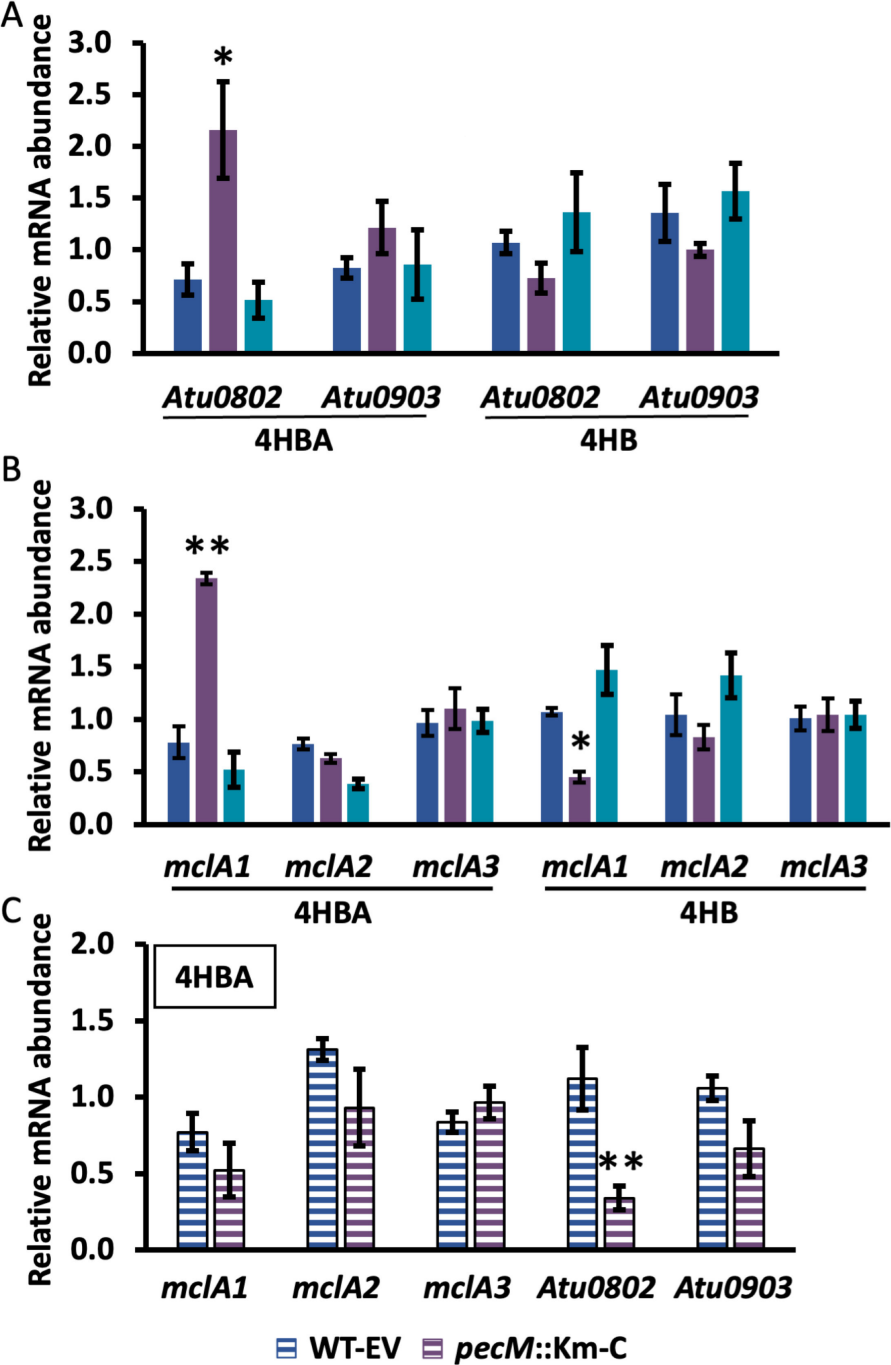
Expression of genes associated with catechol metabolism or chemotaxis during exponential growth in response to 4-hydroxybenzaldehyde (4HBA) or 4-hydroxybenzoate (4HB). (A and B) Gene expression in the identified strains in response to 275 μg mL^−1^ 4HBA or 4HB, as indicated below the graphs. Strains identified as in Fig. 6; blue, WT; purple, *pecM*::Km; cyan, *pecS*::Km. (C). Gene expression in the identified strains in response to 333 μg mL^−1^ 4HBA. Data reflect the mean ± SD from at least three biological replicates. Asterisks reflect statistically significant differences compared to unsupplemented cultures based on a Student’s t-test; *P < 0.05; **P < 0.001).

Aromatic hydrocarbons may be degraded via the catechol branch before converging with the β-ketoadipate pathway; since decarboxylation of protocatechuate would generate catechol, 4HBA and 4HB could potentially also be degraded by this route (9). In *A. fabrum*, the catechol 1,2-dioxygenase that would initiate the conversion of catechol to β-ketoadipate is not identified in the KEGG database. However, Atu0802 and Atu0903 are annotated as catechol 2,3-dioxygenases (CatE), which would divert catechol to the meta-cleavage pathway and lead to the production of pyruvate and acetaldehyde (and not β-ketoadipate) (11). Expression of these two genes in response to 4HBA and 4HB treatment showed that *Atu0802* is modestly induced in the *pecM*::Km strain in response to 4HBA treatment (Fig. 7A). No such induction was observed with 4HB treatment. This result suggests a modest induction of the catechol meta-cleavage branch in response to 4HBA when the compound accumulates sufficiently, as inferred for the *pecM*::Km strain.

*A. tumefaciens* A136 and A348 have been reported to chemotax toward 4HB, which subsequently participates in the induction of virulence genes (8). In *P. putida*, uptake of 4HB through the transporter PcaK promotes chemotaxis by inducing the chemoreceptor gene *pcaY* (7). The best BLAST hit to *P. putida* PcaY is Atu0526 (33% identity and 50% homology), also known as MclA, which has been reported to be a chemoreceptor for formic acid in *A. fabrum* (27). We looked into the expression of the three annotated *mclA* genes, *Atu0526* (*mclA1*), *Atu1912* (*mclA2*), and *Atu3330* (*mclA3*) in response to 4HBA and 4HB treatment. In response to 4HBA, a slight induction of *mclA1* was observed in the *pecM*::Km strain; however, no induction was seen in response to 4HB treatment, which suggests that chemotaxis toward 4HB does not involve induction of either of these *mclA* genes (Fig. 7B). Furthermore, the induction of *Atu0802* and *mclA1* seen in the *pecM*::Km strain in response to 4HBA was lost in the *pecM*::Km-C strain.

### 4HBA is not a ligand for PecS

**Fig 8:**
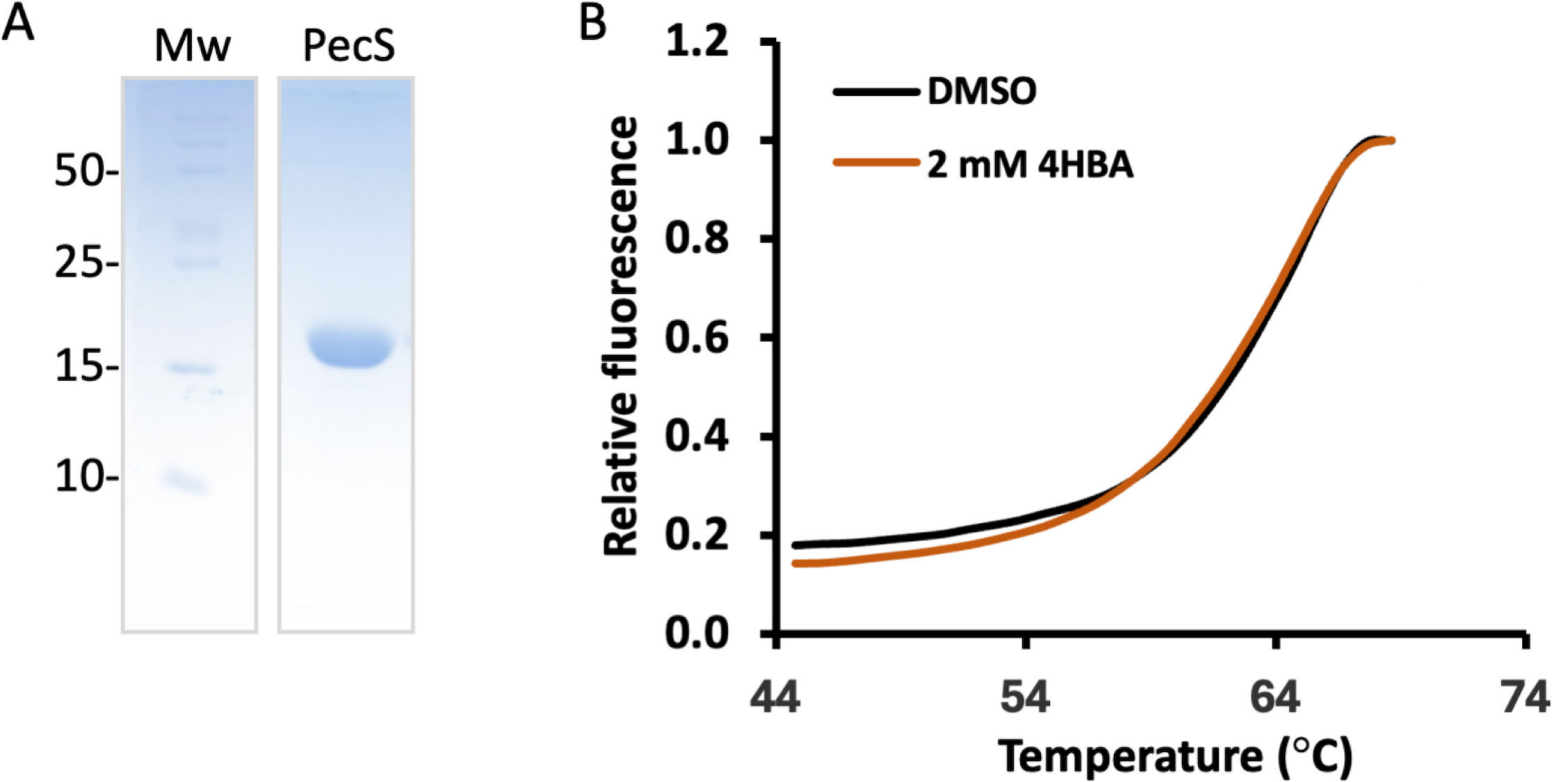
Thermal stability of PecS. (A) Purified PecS. Mw markers are identified at the left in (kDa). Mw markers and PecS were electrophoresed in the same gel, and proteins were visualized by staining with Coomassie Brilliant Blue. The theoretical Mw of His_6_-tagged PecS is 18,923 Da. (B) Thermal unfolding measured by differential scanning fluorimetry of Sypro Orange fluorescence binding to unfolded regions of PecS as a function of temperature. Relative fluorescence of PecS in the presence of DMSO, the solvent for 4HBA (black), and with 2 mM 4HBA (orange). Scans represent the average of three replicates.

The increased *pecM* mRNA accumulation observed on exposure to 4HBA is lost in the absence of PecS (Fig. 6A). As noted above, one possible explanation for this observation is that 4HBA serves as an inducing ligand for PecS, resulting in 4HBA-mediated induction only when PecS is present. We therefore performed *in vitro* assays to address whether 4HBA may be a ligand for PecS. PecS was expressed with an N-terminal His_6_-tag and purified to apparent homogeneity (Fig. 8A). Protein thermal stability was measured in the presence of the fluorescent reporter SYPRO Orange, which binds to hydrophobic regions as protein unfolds as a function of temperature. PecS is quite stable, with Tm 62.5 ± 0.6°C (Fig. 8B, black line). An increase in thermal stability is typically seen when a ligand binds a protein, assuming that the ligand binds preferentially to the native form of the protein. Incubation of PecS with 4HBA (or 4HB or Cm, which were used as controls) had no effect on thermal stability (Table 1).

**TABLE 1:** Tm of PecS with ligands

Ligand	Conc (mM)	Tm (°C)
None		62.5 ± 0.6
4HB	0.4	62.7 ± 0.3
4HBA	0.4	62.4 ± 0.5
4HBA	2.0	62.2 ± 0.5
Cm	0.5	62.6 ± 0.5

**Fig 9:**
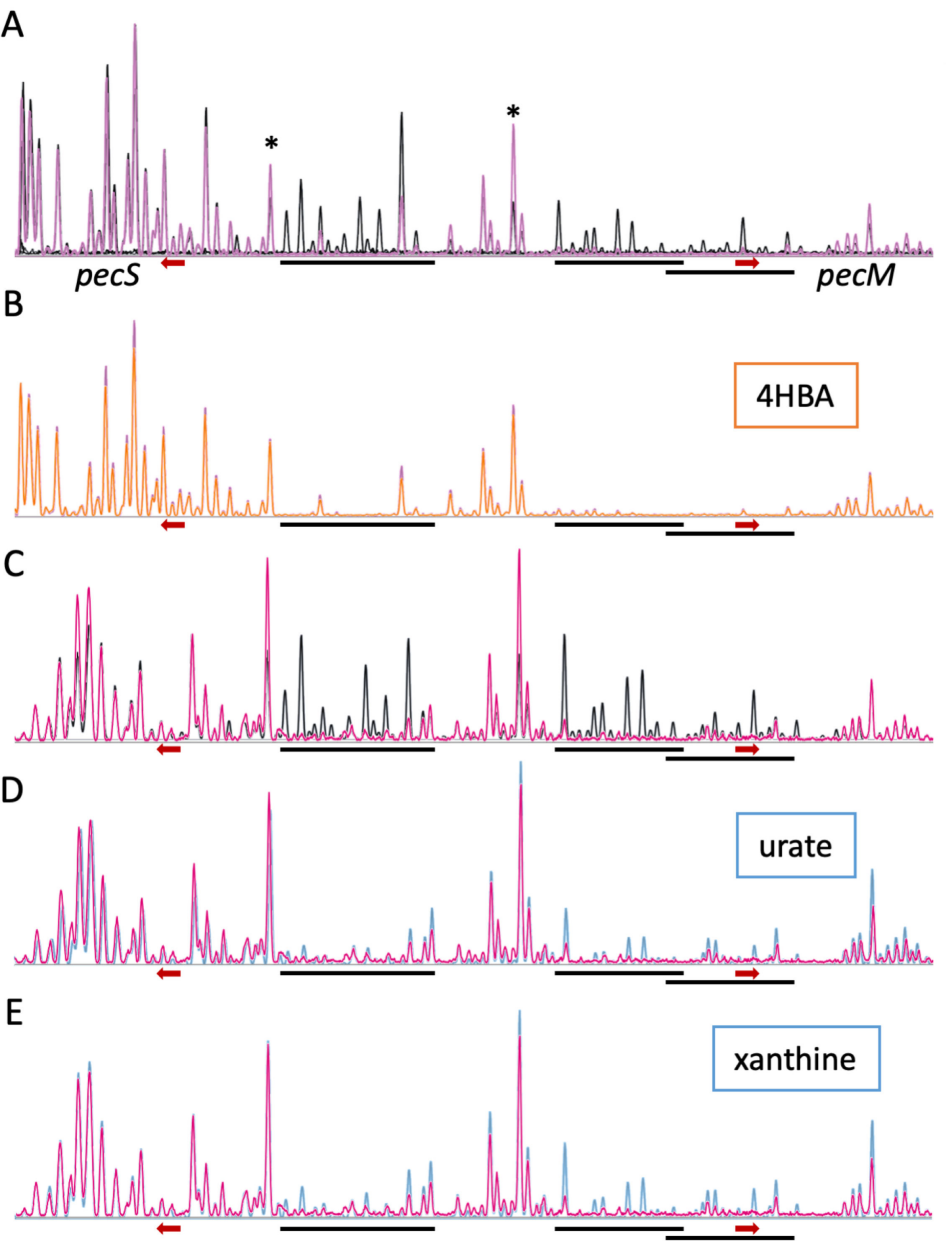
PecS protects regions in both *pecS* and *pecM* promoters. (A) DNase I digestion of DNA containing the *pecS-pecM* intergenic region without (black) or with 43 nM PecS (pink). Asterisks mark hypersensitive cleavage. Palindromic regions within each protected site are marked with a black line (for sequences, see Fig. 2A). The *pecS* and *pecM* start codons are marked with red arrows. (B) Electropherograms of DNA incubated with 43 nM PecS without (pink) and with 250 μM 4HBA (orange). (C) Electropherograms of DNA incubated without (black) or with 86 nM PecS (pink). (D) Electropherograms of DNA incubated with 86 nM PecS without (pink) or with 2.5 mM urate (blue). (E) Electropherogram of DNA incubated with 86 nM PecS without (pink) or with 10 mM Xanthine (blue). Experiments represented in panels (C and E) were performed at high ionic strength, lowering the affinity of all interacting components. Footprints are representative of at least three independent experiments

PecS binds three sites, a single palindromic sequence in the *pecS* promoter and two overlapping sites that span the *pecM* promoter and part of the coding region (Fig. 2A) (18, 28). If 4HBA were a ligand for PecS, it would be expected to lessen or change the PecS footprint. We performed DNase I footprinting using fragment analysis, confirming the previously reported protection of two DNA regions (Fig. 9A). Two sites of enhanced cleavage (marked by asterisks) indicate that PecS imposes a DNA distortion. When PecS was incubated with 4HBA, there was no change whatsoever in the footprint, as evidenced by overlaying electropherograms obtained with PecS in the absence or presence of 4HBA (Fig. 9B). This pattern of protection was consistent, regardless of ligand concentration used. As a control, we examined the footprint in the presence of the known ligands xanthine and urate. Since the purines must be dissolved in 0.4 M NaOH, an elevated concentration of Tris was used to reduce the increase in pH on the addition of the ligand. In this condition, the affinity of PecS for DNA was reduced, and DNase I was less active, requiring a higher concentration of both PecS and the enzyme. The pattern of protection by PecS was unaltered, including the presence of hypersensitive cleavage sites (Fig. 9C). The addition of either urate or xanthine (Fig. 9D and E; blue traces) resulted in recovery of DNase I cleavage within the region protected by PecS. This result indicates a direct interaction between these purines and PecS, consistent with previous findings. Since no evidence for direct interaction between PecS and 4HBA was evident, we surmise that a different 4HBA-responsive transcription factor is responsible for the observed increase in *pecM* and *pecS* mRNA on the addition of 4HBA.

### An assessment of 4HBA toxicity

**Fig 10:**
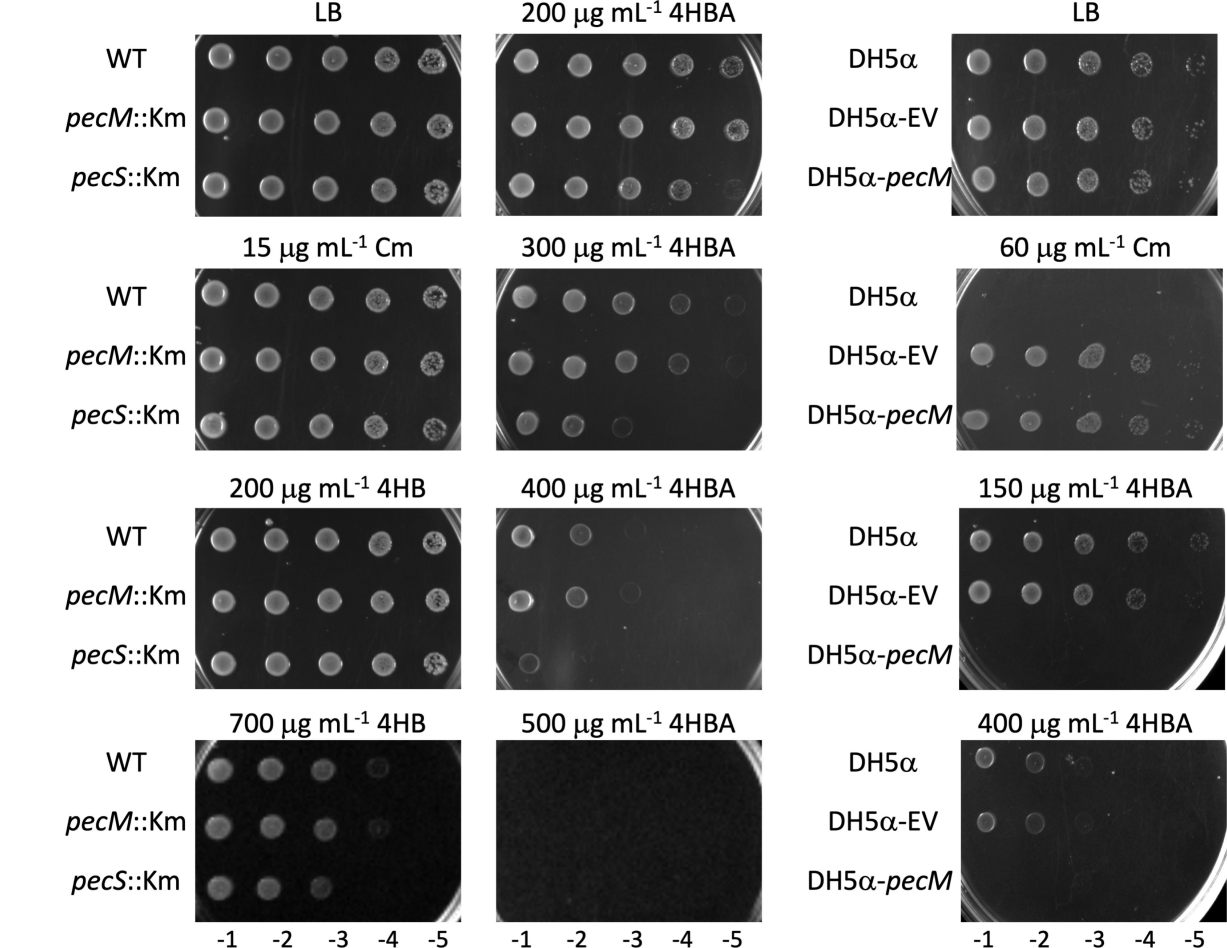
Bacterial growth in the presence of Cm, 4HB, or 4HBA. Cells were grown to an OD ~0.6, and 5 μL of 10-fold serially diluted culture, as identified at the bottom, was spotted on LB plates or LB plates containing the indicated concentrations of Cm, 4HB, or 4HBA. For *A. fabrum*, plates were incubated at 30°C for 3 days before visualization. For *E. coli* DH5α, plates were incubated at 37°C for 12 hours.

Considering that PecM appears to export 4HBA (and marginally Cm), we addressed whether the absence of PecM or overexpression of *pecM* (in *pecS*::Km cells) would alter sensitivity to these compounds using plate assays. As expected, *A. fabrum* was resistant to 15 μg mL^−1^ of Cm, and disruption of either *pecM* or *pecS* had no effect (Fig. 10; left panel).

4HBA and other aromatic aldehydes are prone to interacting with and disrupting the cell membrane due to the hydrophobicity of the aromatic ring and the reactivity of the aldehyde functional group, resulting in cell death, whereas intracellular targets include nucleophilic sites on DNA and proteins (24, 29, 30). Accordingly, the uptake of extracellular 4HBA and its subsequent degradation may avert membrane damage and cell death. All strains were able to grow with 200 µg mL^−1^ of 4HBA, except for the *pecS*::Km strain, which overexpresses *pecM* (Fig. 3E); *pecS*::Km cells exhibited a slight growth defect compared to WT cells (Fig. 10, middle panel). This growth defect became more pronounced at higher concentrations of 4HBA, at which WT and *pecM*::Km strains also exhibited sensitivity. With 500 µg mL^−1^ of 4HBA, no growth was observed for any of the strains. By comparison, a growth defect in the presence of 4HB required a much higher concentration and was observed at 700 μg mL^−1^ (Fig. 10; left panel). This outcome suggests that part of the 4HBA that is taken up by *pecS*::Km cells is rapidly extruded rather than degraded, resulting in disruption of the cell membrane and cell death at higher concentrations of 4HBA. We addressed this interpretation by expressing *pecM* in *Escherichia coli*, which does not encode a PecM homolog, with the expectation that *pecM* expression should sensitize the cells to 4HBA. As seen in Fig. 10 (right panel), this was indeed observed.

## DISCUSSION

### Cellular responses to chloramphenicol

Cm is a broad-spectrum antibiotic that inhibits protein synthesis by binding reversibly to the bacterial 50S ribosomal subunit and preventing peptide bond formation. Resistance to Cm is typically achieved by acetylation of Cm by chloramphenicol acetyl transferase (CAT), as the acetylated drug can no longer bind the ribosomal subunit. CAT enzymes have been divided into three families based on sequence and structural homology, CATA, CATB, and CATC (31). Indeed, the first *catB* gene was described in *A. fabrum* (32). Induction of *catB* expression (Fig. S1A and B) is not the only mechanism by which *A. fabrum* has been shown to deal with Cm. A search for genes associated with responsiveness to acetosyringone, which induces virulence gene expression in *A. fabrum*, led to the identification of the RND efflux pump MexE-MexF-AmeC, the absence of which also resulted in sensitivity to Cm (33). That *mexE* in particular is induced by Cm (Fig. S1C and D) reinforces the role of this efflux pump in the expulsion of Cm. The dual substrate specificity of MexE-MexF-AmeC suggests a relationship between cellular responses to toxic compounds and induction of virulence genes.

Since Cm-responsive genes are induced to a slightly greater extent in *pecM*::Km compared to WT cells (Fig. S1A, C and E), we speculate that PecM contributes modestly to the export of Cm, but only when Cm levels are very high. This contribution is likely not physiologically relevant, as evidenced by comparable sensitivity to Cm for WT and *pecM*::Km cells (Fig. 10A).

Sublethal Cm induces a complex stress response. In *Vibrio parahaemolyticus*, for example, Cm was reported to induce differential expression of 650 genes (34). One of these genes was *HGPRT*, which encodes hypoxanthine-guanine phosphoribosyltransferase, an enzyme that participates in the generation of purine nucleotides through the purine salvage pathway (35). If similar Cm-mediated differential gene expression were to occur in *A. fabrum*, this could readily explain the accumulation of hypoxanthine and xanthine, which are intermediates in purine salvage, as well as urate, which is the first intermediate in purine degradation (Fig. 3). While the reason for the modest accumulation of all purines in *pecS*::Km cells compared to WT and *pecM*::Km cells is unclear, we note that PecS binds both xanthine and urate and that absence of PecS should result in less sequestration of these metabolites.

### Regulation of *pecM* expression

The divergent genes encoding PecS and PecM appear to have been distributed by horizontal gene transfer (20, 36). Integration into existing transcriptional regulatory networks has resulted in PecS adopting distinct regulatory functions in different bacterial species. For instance, PecS is intimately involved in controlling virulence genes in the necrotrophic plant pathogen *D. dadantii* in combination with several other regulatory proteins (37). In the opportunistic pathogen *Klebsiella pneumoniae,* it indirectly regulates genes encoding type 1 fimbriae, which mediate surface attachment (38), and in *Vibrio vulnificus*, it directly controls genes associated with responses to nitrosative stress (39). By comparison, *A. fabrum* PecS appears to exert its regulatory roles prior to infection, affecting processes such as biofilm formation and quorum sensing (19). In all species where PecS has been characterized, it has been shown to repress expression of *pecS* and *pecM* (20). However, in *K. pneumoniae, pecS* and *pecM* expression is also repressed by the stress-responsive two-component regulator CpxR, with PecS-mediated repression only detectable in the absence of CpxR (38). Evidently, PecS has been differentially integrated into regulatory networks where it cooperates with other transcription factors to achieve the requisite control of target genes, including *pecM*.

*A. fabrum* PecS binds a single palindrome in the *pecS* promoter and to two overlapping sites in the *pecM* promoter (Fig. 2A), and these distinct binding modes may underlie the selective induction of *pecS* expression by the PecS ligand xanthine (19). Notably, regulation by 4HBA is opposite, with 4HBA primarily inducing expression of *pecM* in preference to *pecS* (Fig. 6A), suggesting a distinct regulatory mechanism. Since the DNase I footprint is completely unaltered when PecS is incubated with 4HBA (Fig. 9B), and since 4HBA has no effect on the thermal stability of PecS (Fig. 8B), we find it more likely that a separate 4HBA-responsive transcription factor controls *pecM* expression. Since *pecM* is repressed by PecS, this transcription factor likely competes with PecS for binding to the *pecM* promoter, but only in the presence of its co-inducer, 4HBA. By doing so, this factor may either function as an activator or as an anti-repressor. Since 4HBA does not result in elevated levels of *pecM* mRNA in *pecS:*:Km cells (Fig. 6A, cyan bar), the argument could be made that it is an anti-repressor and not an activator. Alternatively, the elevated expression of *pecM* in this strain may result in sufficient export of 4HBA such that too little remains to act as a co-inducer for an activator. This interpretation would predict relatively low-affinity binding to the activator, since sufficient 4HBA clearly remains to elicit induction of genes such as *pcaF* and *pobA*. Regardless of the mechanism or identity of the transcription factor in question, specific induction of *pecM* expression by its substrate is logical, and it would ensure adequate efflux of the toxic 4HBA. The physiological relevance for the participation of a separate transcription factor in the regulation of *pecM* may relate to ensuring sustained or adequate *pecM* expression in the presence of its substrate. If 4HBA were also a ligand for PecS, a comparably increased *pecS* expression might result in rapid accumulation of PecS and consequently in *pecM* repression, possibly defeating the purpose of efficiently exporting 4HBA.

### Cellular responses to 4HBA

PecM exports 4HBA; however, the purpose does not appear to be survival. When PecM is absent, 4HBA accumulates intracellularly (Fig. 5), resulting in elevated induction of genes associated with its degradation (Fig. 6). In this situation, 4HBA possibly derives from degradation of tyrosine (40). Two intermediates in the citric acid cycle (CAC), succinate and α-ketoglutarate, also accumulate markedly (Fig. 5B). Degradation of 4HBA leads to the production of the CAC intermediate succinyl-CoA, which is subsequently converted to succinate, rationalizing the accumulation of succinate when excess 4HBA is degraded. In addition, succinyl-CoA is an inhibitor of α-ketoglutarate dehydrogenase, which converts α-ketoglutarate to succinyl-CoA and is a primary site of control of metabolic flux through the CAC (41). The enzyme glutamate dehydrogenase catalyzes a reversible reaction in which α-ketoglutarate is converted to glutamate, likely accounting for the elevated levels of glutamate in *pecM*::Km cells when levels of α-ketoglutarate increase (Fig. 5B). Evidently, the absence of PecM leads to degradation of excess 4HBA, in turn disrupting metabolic flux through central metabolism, suggesting that PecM serves to maintain cellular homeostasis when *A. fabrum* encounters and degrades 4HBA. Noting that overexpression of *pecM* leads to increased 4HBA sensitivity when exposed to very high concentrations, as seen for *pecS*::Km cells and for *E. coli* harboring plasmid expressing *pecM* (Fig. 10), it is clear that 4HBA export must be carefully tuned to find a balance between optimizing intracellular homeostasis and surviving the effects of extracellular 4HBA.

## MATERIALS AND METHODS

### Bacterial strains, media, and antibiotics

The bacterial strains used are listed in Table 2. The disarmed *A. fabrum* GV3101 was used as wild type (WT). Disruption of *pecS* was previously reported (19). *A. fabrum* strains were grown in Luria Bertani (LB) broth (1% Tryptone, 0.5% yeast extract, and 1% NaCl) with or without antibiotics as indicated at 28°C. Cells harboring the plasmid used for complementation were grown with 60 μg mL^−1^ chloramphenicol (Cm). *E. coli* DH5α harboring a plasmid expressing *pecM* or empty vector was grown in LB with 60 μg mL^−1^ Cm at 37°C. *E. coli* RHO3 was grown in LB with 0.5% NaCl and 200 µg mL^−1^ 2,6-diaminopimelic acid (DAP; MilliporeSigma) at 37°C.

**TABLE 2:** Bacterial strains and plasmids

Strains	Description	Source
*Escherichia coli*		
RHO3	SM10(λpir) *∆asd::FRT* ∆*aphA::FRT* Kan^R^	(42)
BL21(DE3)pLysS	F^–^*omp*T *hsd*S_B_ (r_B_^–^, m_B_^–^) *gal dcm* (DE3) pLysS(Cm^R^)	(43)
DH5α	F^–^ φ80*lac*ZΔM15 Δ(*lac*ZYA-*arg*F)U169 *rec*A1 *end*A1 *hsd*R17(r_K_^–^, m_K_^+^) *pho*A *sup*E44 λ^–^*thi*-1 *gyr*A96 *rel*A1	(44)
*Agrobacterium fabrum*		
GV3101	Rif^R^ wild-type strain of C58C1 harboring pMP90 (pTiC58DT-DNA Gen^R^)	(45)
*pecM::*Km	GV3101 *pecM*::Km	This study
*pecS*::Km	GV3101 *pecS*::Km	(19)
*pecM*::Km-C	*pecM*::Km harboring pBBR-*pecM*	This study
*pecM*::Km-EV	*pecM*::Km harboring pBBRBAD2-Cm	This study
WT-EV	GV3101 harboring pBBRBAD2-Cm	This study
Plasmids		
pKNOCK-Km	Km^R^, suicide vector for *pecM* disruption	(46)
pBBRBAD2-Cm	Expression vector for complementation. P_BAD_ promoter and *araC* gene cloned into pBBR1MCS-1, Cm^R^	(47)
pBBR-*pecM*	pBBRBAD2-Cm carrying the *pecM* gene under the control of its own promoter	This study
pAtPecS	pET100 carrying gene encoding His_6_-PecS	(18)

### RT-PCR

To ascertain whether *pecS-gntR-manR* constitutes an operon, RT-PCR was performed using total RNA isolated from WT cells grown to mid-log phase in LB media. cDNA was prepared with primers complementary to either *manR* or *gntR*, followed by PCR amplification using primers specific to each open reading frame. PCR products were identified by agarose gel electrophoresis and staining with ethidium bromide. For primer sequences, see Table S2.

### Construction of *pecM*-disrupted strain and the complemented strain

To construct a *pecM-*disrupted strain, an ~300 bp *pecM* fragment was amplified using genomic DNA as template with pecMF_SmaI and pecMR_XbaI primers containing flanking restriction sites; for primer sequences, see Table S2. The amplified fragment of *pecM* corresponds to nucleotides 147–466. Purified PCR product was digested with XbaI and SmaI and cloned into the suicide plasmid pKNOCK-Km digested with the same enzymes (46). The ligated product was transformed into *E. coli* RHO3, and cells were plated with 100 µg mL^−1^ kanamycin for selection. A recombinant plasmid was confirmed by sequencing and introduced into *A. fabrum* GV3101 by biparental mating as described (48); donor *E. coli* RHO3(pKNOCK-Km-*pecM*) and *A. fabrum* recipient strain were used in a 1:10 ratio. Both strains were grown separately to an OD_600_ of approximately 0.8 in LB; *E. coli* RHO3 was grown in low salt LB (LSLB, containing 50% less salt) supplemented with 100 µg mL^−1^ kanamycin and 200 μg mL^−1^ DAP. Cells were separately washed twice in LB and resuspended in 50 µL of cold 0.9 g L^−1^ NaCl and incubated at room temperature for 5 min followed by spotting the mixture on a 0.2 µm membrane filter, which was placed on a low salt LSLB agar plate with DAP and kanamycin. After 16 h incubation at 28°C, the conjugation mixture was collected, resuspended in 5 mL of 10 mM MgSO_4,_ and spread on an LB agar plate with 50 µg mL^−1^ rifampicin, 10 µg mL^−1^ gentamycin, and 200 µg mL^−1^ kanamycin and incubated at 28°C for 72 h. Disruption of the *pecM* gene was verified by PCR amplification and sequencing. The *pecM* disruption strain is denoted as *pecM::*Km.

For complementation, the full-length *pecM* gene with its promoter region was amplified with Phusion High-Fidelity DNA Polymerase (NEB) using the primers PecM::Km-CF and PecM::Km-CR, incorporating the restriction sites HindIII and KpnI, using genomic DNA as template. The purified PCR product was cloned into pBBRBAD2-Cm between HindIII and KpnI, and the construct (pBBR-*pecM*) was verified by sequencing. This chimeric vector was used to transform *E. coli* RHO3. Biparental mating was performed using transformed *E. coli* RHO3 and recipient *A. fabrum pecM::*Km, followed by plating on LB agar with kanamycin (200 µg mL^−1^) and Cm (60 µg mL^−1^). Positive clones were confirmed by colony PCR using primer sets CompM1 and CompM2 (Table S2). The complemented mutant strain is referred to as *pecM*::Km-C. Empty vector pBBRBAD2-Cm was transformed into WT and *pecM::*Km *A. fabrum* as a control; these strains are referred to as WT-EV and *pecM::*Km-EV, respectively. pBBRBAD2-Cm, or a plasmid harboring the *pecM* expression construct, was also transformed into *E. coli* DH5α.

### Quantification of purine levels

Cellular concentrations of urate, xanthine, and hypoxanthine were determined using the corresponding Amplex Red Assay kits (Thermo Fisher). A single colony was inoculated in 3 mL of LB media and grown for 48 h at 28°C with shaking; Cm was included where indicated. This culture was used to inoculate 30 mL of LB at a 1:100 dilution. The cells were collected by centrifugation at 1,700 × *g* for 10 min at 4°C when the cultures reached OD_600_ ~1.0 (exponential phase) and 36 h post-inoculation (stationary phase). The cells were washed twice with 2 mL of 1× reaction buffer from the assay kit, and the pellet was stored at −80°C. For the assay, cell pellets were thawed on ice and resuspended in 1× reaction buffer from the assay kit. The cells were lysed by sonication (30% output, pulse mode, total time 10 seconds, 1 minute on ice between each cycle, eight cycles in total). Sonicated samples were centrifuged at 16,000 × *g* for 2 min at 4°C. The lysate was transferred to a clean tube and centrifuged again at 16,000 × *g* for 1 min for further removal of cell debris. The cell-free supernatant was used for the assay following the manufacturer’s instructions; cell-free lysate and the working solution of Amplex Red reagent were added to a 96-well flat-bottom tissue culture plate. The plate was kept at 37°C in the dark for 30 minutes, and the absorbance was measured at 560 nm using a microplate reader. The purine levels were normalized using the total protein concentration measured at 280 nm, and concentrations were calculated based on a standard curve. The results are the mean ± SD from at least three independent experiments.

### Untargeted metabolomics

All samples were extracted by protein precipitation using ice-cold 80% methanol in water with pre-normalization to 300 μg/mL protein concentration. Global metabolomics profiling was performed on a Thermo Q-Exactive Orbitrap mass spectrometer with Dionex Ultimate 3000 UHPLC and autosampler. All samples were analyzed in positive and negative heated electrospray ionization with a mass resolution of 35,000 at m/z 200 as separate injections. Separation was achieved on an ACE C18-PFP, 100 × 2.1 mm, 2 µm column with mobile phase A as 0.1% formic acid in water and mobile phase B as acetonitrile. This is a polar embedded stationary phase that provides comprehensive coverage but does have some limitations in the coverage of very polar species. The flow rate was 350 µL/min. The column temperature was maintained at 25°C. Four microliter was injected for negative ionization mode and 2 µL for positive ionization mode.

For data analysis, MZmine 3.4 (freeware) was used to identify features, deisotope, align features, and perform gap filling to fill in any features that may have been missed in the first alignment algorithm. All adducts and complexes were identified and removed from the data set. The data were searched against an internal retention time metabolite library. Subsequent searches against the Human Metabolome Database (HMDB) were also performed.

Multivariate and univariate statistical analyses were performed to assess clustering of samples based on metabolomic profiles and to identify metabolites with significant changes in abundance between groups. Following univariate statistical analysis of all compounds detected in positive and negative ionization modes independently, the list of compounds was combined between negative and positive ionization modes and reduced to a single representative compound per likely metabolite based on *P*-value (lowest *P*-value compound retained).

### *In vivo* gene expression

A single colony was inoculated in LB media and grown for 48 h at 28°C with shaking, and the culture was used to inoculate fresh LB at a 1:100 dilution. The culture was grown at 28°C in a shaker to an OD_600_ ~0.75. The culture was divided into two flasks; one half was supplemented with ligand, Cm (15 µg mL^−1^) or 4HBA/4HB (275 µg mL^−1^; BeanTown Chemical), and the other half was supplemented with an equal volume of the solvent for the ligand dimethylsulfoxide (DMSO). Both flasks were incubated at 28°C for 45 min with shaking. Cells were harvested, washed with diethyl pyrocarbonate (DEPC)-treated water, re-pelleted, and stored at −80°C. For total RNA extraction, the Monarch Total RNA Miniprep Kit (New England BioLabs) was used. For further DNA contamination removal, Turbo DNase (Thermo Fisher Scientific) was used and absence of DNA verified by PCR. RNA was run in 1.2% of agarose gel for checking integrity and quality, followed by quantification by NanoDrop. The Luna Universal One Step RT-qPCR kit (New England BioLabs) was used for quantifying mRNA transcripts in 20 µL total reaction mixture containing 500 ng DNA per reaction following the manufacturer’s protocol. The plates were run on the Quant Studio 6 real-time PCR system (Applied Biosystems). The primers used for RT-PCR are listed in Table S2. The results were analyzed using Excel. Data represent means (±SD) from at least three biological replicates (each determined from technical triplicates) using the comparative threshold cycle (*C_T_*) method (2^−ΔΔ*CT*^). For experiments to determine differential gene expression in response to Cm, *rpoA* (*Atu1923*) was used as the reference gene, except for strains WT-EV and *pecM*::Km-C, for which *gltB3* (*Atu014*8) was used as the reference gene to determine differential expression of *mexE, mexF, ameC*, and *cmRP*. For experiments involving the addition of 4HB/4HBA, *rarD* (*Atu1068*) was used as a reference gene. The *C_T_* values for the selected housekeeping genes were constant under the conditions used for the respective experiments. Statistical significance was evaluated based on a Student’s *t*-test.

### Sensitivity assays

Sensitivity was assessed using plate assays. *A. fabrum* cultures grown for ~48 hours at 28°C in LB were diluted 1:100 in fresh media and grown to an OD_600_ ~0.6. Five microliter of 10-fold serially diluted culture was spotted on LB plates containing either 15 μg mL^−1^ Cm or the indicated concentrations of 4HBA or 4HB. Plates were incubated at 28°C for 3 days before visualization. For *E. coli* strains, cultures were grown in LB at 37°C and processed similarly, except that plates were incubated at 37°C for 12 hours. Data are representative of at least three replicates.

### Purification of PecS protein

*A. fabrum* PecS was expressed with an N-terminal His_6_-tag and purified as described (18). Briefly, the pET100 plasmid harboring the *pecS* gene was used to transform *E. coli* BL21(DE3)pLysS. Transformed cells were grown overnight in LB supplemented with ampicillin (100 µg mL^−1^) at 37°C and used at a 1:100 dilution to inoculate fresh LB media with ampicillin. The culture was grown with shaking at 37°C to an OD_600_ of ~0.5, and protein expression was induced with 0.5 mM isopropyl β-D-1-thiogalactopyranoside for 2 h. Cells were pelleted and stored at −80°C. PecS was purified using Ni-NTA Agarose (Sigma-Aldrich) chromatography. Protein was eluted with a linear gradient of imidazole from 20 mM to 250 mM. The pooled fractions were buffer exchanged for removal of imidazole at 4°C using centrifugal filters (Amicon Ultra-15). Purity was verified by electrophoresis in a 15% SDS-PAGE gel, followed by visualization with Coomassie Brilliant Blue. Concentration was determined spectrophotometrically using an extinction coefficient of 8,480 M^−1^ cm^−1^ for the His6-tagged PecS.

### Protein thermal shift assay

Protein thermal shift assay was carried out as described (18, 49). PecS (3.6 µM) was added to a buffer containing 50 mM Tris (pH 8.0), 100 mM NaCl, and 5× Sypro Orange (Invitrogen) as fluorescence reporter in a final volume of 25 µL. For ligand binding assays, the reaction mixture was supplemented with DMSO or 2 mM 4HBA in DMSO. The change in fluorescence intensity upon binding of the reporter molecule to unfolded protein was measured using a Quant Studio 6 Real Time PCR instrument (Applied Biosystems, filter: SYBR Green) over a temperature range of 4°C to 90°C at a scan rate of 1°C increment per 45 s. The total fluorescence yield measure was corrected using reactions without protein as a control. Data were analyzed using Protein Thermal Shift software (Applied Biosystems) for determination of melting temperature (Tm) by fitting the data to a Boltzmann sigmoidal equation. The Tm is reported as the mean ± SD of triplicate measurements.

### DNase I footprinting

DNA representing 96 bp spanning the intergenic region between *pecS* and *pecM* start codons as well as 32 bp of *pecS* coding sequence and 62 bp of *pecM* coding sequence (total 190 bp) was PCR amplified with primers pecO-Fw that was labeled at its 5′-end with carboxyfluorescein (6-FAM) and pecO-Rv using genomic DNA as template; for primer sequences, see Table S2. DNase I footprinting was performed essentially as described (50). In brief, 50 ng DNA was incubated for 10 min with or without PecS (43 nM) in binding buffer (25 mM Tris pH 8, 0.1 mM EDTA, 100 mM NaCl, 0.05% Brij, 10 mM DTT, 2% glycerol) in a final volume of 10 µl, followed by the addition of 0.02 units of DNase I. A reaction mixture with high ionic strength (binding buffer containing 250 mM Tris, pH 8.0) was used to lessen the increase in pH upon the addition of the ligands urate and xanthine, which were dissolved in 0.4 M NaOH. In these reactions, 86 nM PecS was used as the affinity of PecS for the DNA is reduced at higher ionic strength, and the DNase I concentration was increased to 0.2 unit per reaction. The high concentrations of urate and xanthine reflect the reduced affinity of the ligand for PecS at high ionic strength as well as the poor solubility of both ligands, which precipitate at neutral pH. In all reactions where a ligand was added, DNA and ligand were mixed first in binding buffer, and protein was added last. All reactions were incubated at room temperature for precisely 3.5 min and then stopped by bringing the EDTA concentration to 7.5 mM. The digested DNA was purified and dissolved in 10 µl Hi-Di formamide. One microliter of dissolved DNA was combined with 1 µL of LIZ 600 ladder (ABI-Life Technologies) and Hi-Di formamide to a final volume of 10 µL and subjected to fragment analysis using an ABI_DS33 analyzer. Microsatellite Analysis Software (ThermoFisher Cloud) was used to analyze sequencing data. Footprints are representative of at least three replicates.

## Data Availability

All data are available in this manuscript and the supporting documentation.

## SUPPLEMENTAL MATERIAL

Figures S1 and S2 jb.00150-25-s0001.pdf Gene expression in response to chloramphenicol or 4HBA.

Table S1 jb.00150-25-s0002.xlsx Untargeted metabolomics dataset.

Table S2 jb.00150-25-s0003.pdf Primer sequences.

ASM does not own the copyrights to Supplemental Material that may be linked to, or accessed through, an article. The authors have granted ASM a non-exclusive, world-wide license to publish the Supplemental Material files. Please contact the corresponding author directly for reuse.
