# Supplementary material for: The Agrobacterium fabrum efflux pump PecM is produced in response to the plant exudate 4-hydroxybenzaldehyde to avoid disruption of central metabolism
Source: J Bacteriol. 2025 Jun 13;207(7):e00150-25. doi: 10.1128/jb.00150-25 (PMC12288460; doi:10.1128/jb.00150-25)
Supplement: Figures S1 and S2 — Gene expression in response to chloramphenicol or 4HBA. [file jb.00150-25-s0001.pdf]

The *Agrobacterium fabrum* efflux pump PecM is produced in response to the plant exudate 4-hydroxybenzaldehyde to avoid disruption of central metabolism

Arpita Ghosh and Anne Grove
Department of Biological Sciences, Louisiana State University, Baton Rouge, LA 70803, USA

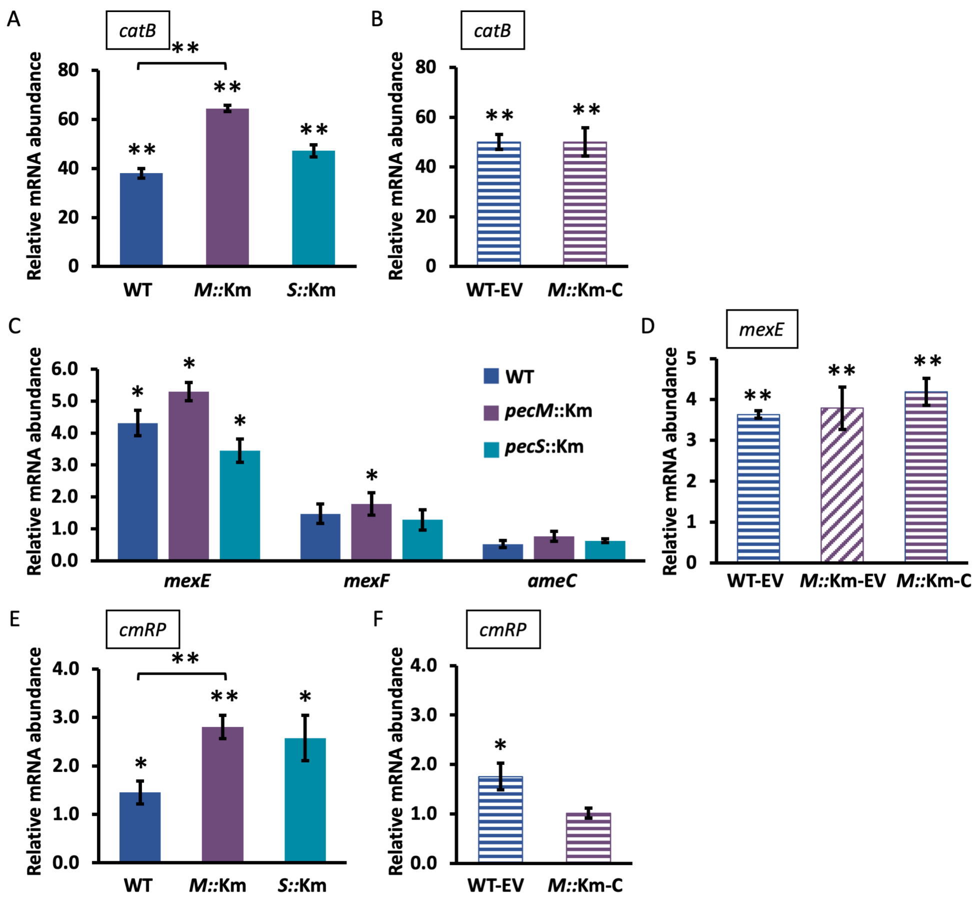

Supplemental Figure S1. Gene expression in response to chloramphenicol (Cm). A. Expression of *catB* in WT (blue), *pecM*::Km (*M*::Km; purple), and *pecS*::Km (*S*::Km; cyan) cells in response to 15 µg mL^{-1} Cm. B. Expression of *catB* in WT-EV and *pecM*::Km-C (*M*::Km-C) grown with 60 µg mL^{-1} Cm. C. Expression of *mexE, mexF*, and *ameC* in the identified strains in response to 15 µg mL^{-1} Cm. D. Expression of *mexE* in WT-EV, *pecM*::Km-EV (*M*::Km-EV) and *pecM*::Km-C (*M*::Km-C) grown with 60 µg mL^{-1} Cm. E-F. Expression of *cmRP* in the identified strains in response to 15 µg mL^{-1} (E) or 60 µg mL^{-1} (F) Cm. G. Expression of *pecM* in *pecS*::Km (*S*::Km) and *pecM*::Km-C (*M*::Km-C). Data reflect the mean ± SD from at least three biological replicates with mRNA isolated during exponential growth. Asterisks reflect statistically significant differences compared to unsupplemented cultures unless identified otherwise; based on a Student's t-test; *, $p<0.05$; **, $p<0.001$).

Chloramphenicol acetyl transferase B (*catB*, Atu4738) is inducible by Cm and regulated by translation attenuation, with increased levels of *catB* mRNA detectable on addition of Cm proposed to be due to an increase in the stability of the mRNA (1, 2). As expected, Cm induced *catB* in all the strains tested, with the greatest accumulation of *catB* mRNA in the *pecM*::Km strain; this would be consistent with the inference that PecM contributes to Cm resistance by exporting this antibiotic, resulting in higher cellular levels of Cm in the *pecM*::Km strain. For WT-EV and *pecM*::Km-C strains, which were exposed to higher concentration of Cm (60 µg mL^{-1}), the level of induction of *catB* was only modestly greater than that observed in WT, indicating that maximal induction of *catB* was almost achieved at the lower Cm concentration (15 µg mL^{-1}).

A resistance-nodulation-division (RND) type efflux pump MexE-MexF (Atu2482-Atu2483) and the outer membrane component AmeC (Atu2552), encoded by a different efflux pump operon, was shown to participate in Cm resistance as evidenced by Cm hypersensitivity of *mexE* and *ameC* mutants (3). These authors did not explore induction of the respective genes by Cm. We investigated the expression of *mexE* (encoding the periplasmic adapter subunit), *mexF* (encoding the permease), and *ameC* in response to Cm treatment. The results showed that *mexE* was significantly induced in WT and both mutants, with slightly higher expression in *pecM*::Km. The expression of *mexF* was only marginally increased in *pecM*::Km, while *ameC* mRNA levels were not increased. The accumulation of *mexE* mRNA in WT-EV, *pecM*::Km-EV, and *pecM*::Km-C strains exposed to a higher concentration of Cm was similar to that observed in WT, suggesting that the lower concentration of Cm effectively results in maximal induction of *mexE*. The stoichiometry of the well-characterized *E. coli* RND type efflux pump AcrA-AcrB-TolC, in which the AcrA subunit is the periplasmic adapter, has been reported. AcrB (permease) and TolC (outer membrane channel) are homotrimers, and they are connected by AcrA, generating an AcrA:AcrB:TolC stoichiometry of 6:3:3 (4, 5). This model rationalizes the requirement for expressing more of the periplasmic adapter subunit compared to other components of the efflux pump.

The gene encoding a major facilitator superfamily (MFS) efflux pump annotated as CmRP (chloramphenicol resistance protein; Atu4292) was modestly induced, particularly in *pecM*::Km cells. A modest induction was also seen in WT-EV, whereas no accumulation of *cmRP* mRNA was detected in *pecM*::Km-C, which overexpresses *pecM*.

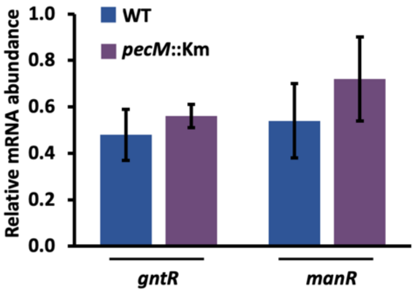

Supplemental Figure S2. Expression of *gntR* and *manR*. Gene expression in the identified strains in response to 275 μg mL^{-1} 4HBA during exponential growth. Data reflect the mean ± SD from three biological replicates.

References

1. Tennigkeit J, Matzura H. 1991. Nucleotide sequence analysis of a chloramphenicol-resistance determinant from *Agrobacterium tumefaciens* and identification of its gene product. Gene 98:113-6.
2. Rogers EJ, Rahman MS, Hill RT, Lovett PS. 2002. The chloramphenicol-inducible *catB* gene in *Agrobacterium tumefaciens* is regulated by translation attenuation. J Bacteriol 184:4296-300.
3. Binns AN, Zhao J. 2020. The MexE/MexF/AmeC Efflux Pump of *Agrobacterium tumefaciens* and Its Role in Ti Plasmid Virulence Gene Expression. J Bacteriol 202:e00609-19.
4. Tikhonova EB, Yamada Y, Zgurskaya HI. 2011. Sequential mechanism of assembly of multidrug efflux pump AcrAB-TolC. Chem Biol 18:454-63.
5. Xu Y, Lee M, Moeller A, Song S, Yoon BY, Kim HM, Jun SY, Lee K, Ha NC. 2011. Funnel-like hexameric assembly of the periplasmic adapter protein in the tripartite multidrug efflux pump in Gram-negative bacteria. J Biol Chem 286:17910-20.
