# Supplementary material for: The Agrobacterium fabrum efflux pump PecM is produced in response to the plant exudate 4-hydroxybenzaldehyde to avoid disruption of central metabolism
Source: J Bacteriol. 2025 Jun 13;207(7):e00150-25. doi: 10.1128/jb.00150-25 (PMC12288460; doi:10.1128/jb.00150-25)
Supplement: Table S2 — Primer sequences. [file jb.00150-25-s0003.pdf]

The *Agrobacterium fabrum* efflux pump PecM is produced in response to the plant exudate 4-hydroxybenzaldehyde to avoid disruption of central metabolism

Arpita Ghosh and Anne Grove
Department of Biological Sciences, Louisiana State University, Baton Rouge, LA 70803, USA

Supplemental Table S1. Primer sequences.

Name	Sequence (5' to 3')	Purpose
pecMF_SmaI	CCGGTATCCCGCGGCTGCTTCTCGT	*pecM* disruption
pecMR_XbaI	GGCAAGACCCGCGGCAATCTAGATACCATCTAGTGCC	
pecMC_F	CACTCATCGTTATCGGTCTGTC	Confirmation of *pecM* disruption
pecMC_R	CTACGTGTTCCGCTTCCTTTA	
PecM::Km-CF	CATTGCGCGAGAAGCTTGTCGACGT	Amplification of *pecM* for complementation
PecM::Km-CR	GGCTCCACGGTACCTGTCATGCGC	
CompM1_F	GCGTTTAAGGGCACCAATAAC	Confirmation of *pecM* complementation
CompM1_R	TCGCCCTATAGTGAGTCGTATTA	
CompM2_F	TATCCTGCTGCTGCTTCTC	Confirmation of *pecM* complementation
CompM2_R	GGCAACACCGATACCATCTA	
pecS1_F	GGACCACGTCGACCATATTC	*pecS* cDNA generation and PCR
pecS1_R	CATGGTCATAGCCAGCAGAT	pecS PCR
gntR1_F	ACTTTCGCTCTCGCAACA	*gntR* cDNA generation and PCR
gntR1_R	GAGCGTTACGGTTCTGACTT	*gntR* PCR
manR1_F	CCATACGGATGAGGGCATTAC	*manR* cDNA generation and PCR
manR1_R	AGGTATTGTAGGTGCGGATTTC	*manR* PCR
pecO-Fw	[6-FAM]CATTGCGCGAGAATTCGGTCGA	Footprinting
pecO-Rv	CAGATGGCGAATTCAAGTGCTGTGA	
rpoA_F	GAGCGTGGTTTCGGTCTTAC	*rpoA* gene expression (HKG)
rpoA_R	CATCTTGATGGCGATTTCCT	
rarD_F	AGAACCGCGCTGAGAAAT	*rarD* gene expression (HKG)
rarD_R	GCACCGATCGACCAGATATAG	
gltB_F	CCAGCGTTATGTCGCCTATT	*gltB3* gene expression (HKG)
gltB_R	CATAACCCATGGCCGAGATT	
pecM_F	TGCTGGTCACAGCACTTG	*pecM* gene expression
pecM_R	GGCGACATGCAACGGATA	

pecS_F	CAGCGTCTTGATATCGCTGA	*pecS* gene expression
pecS_R	CTCGAAGTCCTGCAGAAACC	
gntR_F	TGCGATGTCGCTGATGTT	*gntR* gene expression
gntR_R	GAGCGTTACGGTTCTGACTT	
manR_F	GTGGAGACCTATGTGCATGAAT	*manR* gene expression
manR_R	GGAATCCGAGATAACCGACAAG	
catB_F	CACTCGACAAGCAGGTGAA	*catB* gene expression
catB_R	AACTGTGGCCGTGGTAATAG	
mexE_F	TCGGCAGGATCGAAATCAC	*mexE* gene expression
mexE_R	GCTTGCATAGATCGGGTTGA	
mexF_F	CGTCATTCTCTTTCTCCAGACC	*mexF* gene expression
mexF_R	GGAGAAACCGAAGGCATACA	
ameC_F	GTATTCCGGCCGACCTTATT	*ameC* gene expression
ameC_R	CAGCGTGATGCTTGGATAGA	
cmRP_F	GGCCTCATCGTCAGTGTTTAT	*cmRP* gene expression
cmRP_R	CAGAAGCGGCTTTCTTTCAATC	
pcaF_F	CGAGGCGGAACTGATGATT	*pcaF* gene expression
pcaF_R	CCGATGGTGGTATCGTAGATTT	
pcaK_F	CGATCTTCATGCGCTTCCT	*pcaK* gene expression
pcaK_R	TGCGTTGCAGACCATAACT	
pcaR_F	CCAGAGCACTTCCGTTTCTATTC	*pcaR* gene expression
pcaR_R	ATCAGCGCAATCGACATCAC	
pchA_F	GATATTCGTCTCCTCACCTTCAC	*pchA* gene expression
pchA_R	GTTCCAGCCCGAGTTTCTT	
pobA_F	GGTCAACCACGAACTCATCTAC	*pobA* gene expression
pobA_R	GGTCATCCAGCGAACATTGA	
pobR_F	TTGCCGATCCCGCTTATTT	*pobR* gene expression
pobR_R	GCCCGTATCGCCCATTT	
Atu 0802_F	GCCTTAACGACACCGATACA	*Atu0802* gene expression
Atu 0802_R	CCGAGTACTGAGCGGTAATAAT	
Atu 0903_F	TGCCACCCATCACAATATCC	*Atu0903* gene expression
Atu 0903_R	GCGATAGACCTCGATACCATTG	
mclA1_F	CATTCGCTTGAAGGACAACATC	*mclA1* gene expression
mclA1_R	AGGTCACCATCCACCATTTC	
mclA2_F	TGGCGTGACGCGTATTT	*mcl2* gene expression
mclA2_R	TTCATGCTCTGGCGGATAAG	

mclA3_F	GACAGACCTTGCGGAACAT	*mclA3* gene expression
mclA3_R	GTTTCTCTACGCCCTCGATAAC	
